# The evolving burden of heart failure in China: a 34-year subnational analysis of trends and causes from the Global Burden of Disease Study 2023

**DOI:** 10.1186/s40779-025-00650-y

**Published:** 2025-10-09

**Authors:** Xuan Yang, Zhen-Ping Zhao, Yu Shi, Gui-Yuan Han, Yan Xu, Yi-Chong Li, Mai-Geng Zhou

**Affiliations:** 1 Department of Epidemiology, School of Public Health, Cheeloo College of Medicine, Shandong University, Jinan, 250012 China; 2 National Clinical Research Center for Cardiovascular Diseases, Heart Failure Ward, Fuwai Hospital Chinese Academy of Medical Sciences, Shenzhen, 518057 Guangdong China; 3 National Center for Chronic and Noncommunicable Disease Control and Prevention, Chinese Center for Disease Control and Prevention, Beijing, 100050 China; 4 Clinical Research Center, Shenzhen Medical Academy of Research and Translation, Shenzhen, 518048 Guangdong China

**Keywords:** Heart failure (HF), China, Global Burden of Diseases, Epidemiology

## Abstract

**Background:**

Heart failure (HF) continues to be a public health issue in China with population aging and increasing disease drivers. The burden, spatial patterns, temporal trends, and underlying causes of HF in China at subnational levels remain inadequately understood. This study aims to assess the disease burden and causes of HF, and their regional disparities in China from 1990 to 2023.

**Methods:**

Utilizing the estimates from the Global Burden of Diseases, Injuries, and Risk Factors Study (GBD) 2023, we assessed the prevalence and years lived with disability (YLDs) of HF and their trends in China at both national and subnational levels. Estimates were also compared by disease severity, sex, age group, cause, and region.

**Results:**

In 2023, China had an estimated 14.3 million HF cases, marking a significant 208.4% increase over the past three decades. Ischemic heart disease (IHD) has become the top cause of HF, compared with 1990, with hypertensive heart disease (HHD) at the top. Alongside chronic obstructive pulmonary disease (COPD), these three leading causes accounted for 77.4% of all HF cases. The burden of HF due to IHD and COPD exhibited distinct regional patterns and substantial disparities: regions in northern China exhibited notably higher age-standardized YLDs rates for HF due to IHD, while provinces in the western regions faced the highest burden from COPD. Six provinces with the highest tertile of YLDs rates in 2023 also experienced the most pronounced increases between 1990 and 2023.

**Conclusions:**

HF remains a significant public health challenge in China, with a marked increase in prevalence over the past three decades. The substantial regional variations highlight the need for targeted and region-specific public health strategies. Enhanced nationwide efforts should be made to reduce the geographical disparities in the burden of HF.

**Supplementary Information:**

The online version contains supplementary material available at 10.1186/s40779-025-00650-y.

## Background

Heart failure (HF) is a complex clinical syndrome representing the advanced stage of various heart diseases and has become a major public health concern in China and globally [1]. The Global Burden of Diseases, Injuries, and Risk Factors Study (GBD) 2021 estimated that over 55 million people were living with HF worldwide in 2021 [2]. The overall prevalence of HF is expected to increase substantially due to population aging, increasing disease drivers, and the advances in medication and treatment strategies that prolong the lives of individuals with HF [3, 4]. Besides the high morbidity, mortality, and readmission rate, as well as poor outcomes and quality of life [5], HF also imposed a huge economic burden on the healthcare systems and individuals. The overall health expenditure was estimated at $108 billion in 2012 worldwide [6], and the annual cost per capita for HF inpatients in China amounted to $4406.8 [7]. In the past three decades, China has experienced nearly one-third of the global increase in patients with HF [8]. Meanwhile, the increasingly prevailing cardiovascular risk factors and an aging population signal an impending HF epidemic in China and its provinces [9]. Therefore, it is essential to understand the epidemiological pattern of HF to guide the development and implementation of evidence-based prevention and management strategies. Moreover, China has launched the Healthy China 2030 health policy to strengthen the prevention and control of cardiovascular diseases (CVDs), aiming at reducing CVD mortality in China by 190.7 per 100,000 population by the year 2030 [10]. Timely and precise estimation of HF burden in China is crucial to guide the government to improve the prevention and treatment of HF, therefore reducing CVD mortality and achieving the Healthy China 2030 target.

HF is the common final pathway of most CVDs, making it essential to understand its underlying causes. Although most CVDs share common risk factors, treatment and management strategies differ based on specific causes [11]. Identifying HF causes in China can inform effective public health and clinical intervention strategies, improving management and prognosis. Accurately estimating HF burden and trends by cause is fundamental for supporting tailored health policy development and enhancing cardiovascular health nationwide.

Previous studies have estimated the global burden of HF, including China [2, 8], while geographic variations of HF burden across provinces in China remain unclear. Moreover, GBD 2017 identified that ischemic heart disease (IHD), hypertensive heart disease (HHD), and chronic obstructive pulmonary disease (COPD) were the top three global causes of HF, accounting for almost 75% of the age-standardized prevalence rate (ASPR) of HF [8]. However, a systematic analysis of sociodemographic distributions and causes of HF across provinces in China has not yet been conducted. Analyzing HF causes at subnational levels could guide region-specific targeted policies and interventions to prevent these causes from progressing into HF.

In this study, we extracted data from GBD 2023 to analyze the prevalence and disability burden of HF in China and its provinces from 1990 to 2023. We aimed to inform government policymaking by identifying the main cause of HF and priority regions for prevention and management, thereby guiding the medical resource allocations, improving regional performance, and promoting health equity.

## Methods

### Overview

We followed the analytic framework of the GBD study, as extensively detailed elsewhere [12]. Briefly, the GBD 2023 study consolidated global data on incidence, prevalence, and mortality to provide systematic and up-to-date assessments of the fatal and nonfatal health loss for 375 diseases and injuries in 204 countries and territories from 1990 to 2023. The study metrics of HF in China encompassed prevalence numbers, all-age rates, age-standardized rates, and years lived with disability (YLDs) for overall HF and varied causes. Our analysis utilized data from subnational estimates from GBD 2023, covering 31 mainland provinces, autonomous regions, municipalities, and the Hong Kong and Macao Special Administrative Regions (SAR) of China. The localized assessment of disease burden in China constituted an essential part of the GBD initiative, offering comprehensive and comparative estimates of health and health loss at national and provincial levels [12], Employing Disease Modelling Meta-Regression version 2.1 (DisMod­MR 2.1), a Bayesian meta-regression tool, GBD 2023 integrated data on HF incidence, prevalence, remission, and mortality, yielding consistent estimations for different demographic groups and years. This study adheres to the Guidelines for Accurate and Transparent Health Estimates Reporting (GATHER) statement [13].

### Definitions

The GBD study defined HF cases according to a clinical diagnosis of HF using structured criteria such as the Framingham or European Society of Cardiology criteria. This definition could capture the American College of Cardiology/American Heart Association Stage C and D, consisting of both patients who have been diagnosed with HF but are currently asymptomatic and those who are currently symptomatic [1]. To estimate YLDs, HF was further categorized into 4 severity levels: treated, mild, moderate, and severe HF (Additional file 1: Table S1). The methods for severity distribution and estimation of disability weights have been described elsewhere [12]. Diseases causally linked to HF were identified based on a literature review, including rheumatic heart disease (RHD), IHD, HHD, COPD, and the other 36 causes. A full list of causes and their corresponding International Classification of Diseases (ICDs) codes can be found in the Additional file 1: Table S2. The primary data sources on non-fatal estimates of HF in China were derived from systematic reviews of national surveys, published literature, and registries. These data sources were obtained from the Global Health Data Exchange website (https://ghdx.healthdata.org/) [14–20].

### Statistical analysis

We analyzed the prevalence, YLDs, and 40 underlying causes of HF in 1990 and 2023, examining trends across China and its provinces. Comparisons were made with the global average and members of the Group of 20, excluding the European Union (G20, an international economic cooperation forum composed of 20 major economies). We calculated the estimated annual percentage change (EAPC) to quantify the temporal trends in HF burden from 1990 to 2023 with a linear regression model: $y = \alpha + \beta x + \varepsilon$, where $y$ represents the ln (age-standardized rate), $x$ is the calendar year, and $\varepsilon$ is the error term. The EAPC was calculated as $EAPC = 100 \times \left[ {\exp \left( \beta \right) - 1} \right]$. Considering the uncertainty in GBD estimates, we calculated the 95% uncertainty intervals (UIs) of EAPC using Monte Carlo simulation analysis. Random draws of 1000 samples were obtained from the distributions of age-standardized rates for HF prevalence or YLDs rates, and an EAPC was determined for each draw. The 95% UIs represented the 2.5th and 97.5th percentiles of the ordered EAPC estimates, offering a robust measure of variability. The final estimates were the mean estimates across 1000 draws. A statistically increasing trend of age-standardized rate occurs when both 95% UI limits exceed 0; a decreasing trend when both limits of the 95% UIs are below 0; and a constant trend when the 95% UIs contain 0, indicating no significant change over time. We assessed the contribution of each underlying cause to the total HF burden and compared rankings based on prevalence and YLDs between 1990 and 2023. Estimates were presented as absolute numbers, all-age rates, and age-standardized rates per 100,000 population (with 95% UIs) by disease severity, age, sex, year, cause, and location. Age-standardized rates of estimates were calculated by using the GBD world standard population, with 95% UIs derived from the 2.5th and 97.5th percentile values across 250 draws from the estimate process. Compared with previous GBD iterations, GBD 2023 reduced the number of draws from 500 to 250 because simulation tests showed that this reduction would not affect final estimate results and their uncertainty [12]. YLDs were calculated by multiplying the sequela-specific prevalence of HF by the corresponding disability weights.

Provinces were further classified based on patterns of HF disability burden increase, using a quadrant analysis of EAPC and YLDs rates. We empirically calculated the 33rd and 66th percentiles (lower and upper terciles) as cut-off points in both metrics to classify provinces into nine categories. All analyses were performed using R software (version 4.3.2).

## Results

### National level and compared with the G20

In 2023, there were 14.3 million (95% UI 12.3–16.4) HF cases [prevalence rate 998.4 (95% UI 859.4–1145.2) per 100,000 population], of which 5.2 (95% UI 4.5–6.1) million were treated HF, 2.7 million (95% UI 2.0–3.5) were mild HF, 1.7 million (95% UI 1.3–2.3) were moderate HF, 4.6 million (95% UI 3.9–5.4) were severe HF, and 1.4 million (95% UI 1.0–2.0) YLDs due to HF in China (Additional file 1: Tables S3, S4). The number of prevalence cases for overall HF in China represented over one-quarter of the global total [56.1 million (95% UI 50.0–62.3)] (https://vizhub.healthdata.org/gbd-results/). The absolute number of HF cases in China increased by 208.4% (95% UI 188.5–224.4) and YLDs increased by 207.5% (95% UI 189.5–223.1) between 1990 and 2023 (Additional file 1: Tables S3, S4). In 2023, the ASPR of HF ranked 11th among the G20 countries, at 677.0 (95% UI 591.5–766.2) per 100,000 population, higher than the global average of 641.3 (95% UI 573.9–708.6). From 1990 to 2023, the ASPR increased by 9.4% (95% UI 4.1–14.1), obviously surpassing the global increase of 2.7% (95% UI − 0.6 to 5.8). EAPC of the ASPR in China [0.30% (95% UI 0.09–0.52)] was significantly higher than the global average [0.09%, (95% UI − 0.08 to 0.28)]. In 2023, the age-standardized YLDs rate of HF in China ranked 8th among the G20 countries. The highest percentage change of age-standardized YLDs rate was seen in India [10.2, (95% UI 6.9–13.8)], followed by China [9.1, (95% UI 4.0–13.7)] (Table 1).

**Table 1:** Age-standardized prevalence and YLDs rate of heart failure in 2023, and their percentage changes and EAPC from 1990 to 2023, 19 member countries of the G20 (the 20th member is the European Union), and the world

Location	Prevalence			YLDs		
	ASR [per 100,000 population (95% UI)]	Percentage change (95% UI)	EAPC [% (95% UI)]	ASR [per 100,000 population (95% UI)]	Percentage change (95% UI)	EAPC [% (95% UI)]
Argentina	715.3 (626.0–815.6)	− 25.6 (− 32.6 to − 17.6)*	− 0.89 (− 1.18 to − 0.57)*	60.9 (42.3–82.7)	− 18.5 (− 26.5 to − 10.9)*	− 0.62 (− 1.24 to 0.03)
Australia	793.5 (706.7–875.1)	− 2.3 (− 11.4 to 8.4)	− 0.35 (− 0.56 to − 0.11)*	74.4 (51.3–103.7)	− 1.9 (− 12.3 to 9.1)	− 0.34 (− 0.99 to 0.38)
Brazil	695.2 (623.2–765.3)	− 14.5 (− 21.4 to − 7.9)*	− 0.45 (− 0.65 to − 0.23)*	61.2 (42.2–85.5)	− 7.9 (− 14.3 to − 2.5)*	− 0.23 (− 0.85 to 0.42)
Canada	900.4 (779.4–1032.0)	0.7 (− 8.0 to 9.4)	− 0.08 (− 0.32 to 0.17)	84.5 (57.9–118.6)	1.6 (− 7.8 to 11.6)	− 0.05 (− 0.72 to 0.66)
China	677.0 (591.5–766.2)	9.4 (4.1–14.1)*	0.30 (0.09–0.52)*	66.0 (45.6–93.2)	9.1 (4.0–13.7)*	0.26 (− 0.38 to 0.96)
France	1137.8 (990.2–1287.4)	3.5 (− 2.2 to 10.3)	− 0.14 (− 0.33 to 0.07)	108.7 (75.3–155.7)	3.8 (− 3.0 to 11.1)	− 0.11 (− 0.76 to 0.59)
Germany	697.4 (609.3–790.4)	4.0 (− 1.9 to 10.6)	0.58 (0.38–0.80)*	65.4 (45.4–93.9)	3.9 (− 3.0 to 10.9)	0.58 (− 0.07 to 1.27)
Global	641.3 (573.9–708.6)	2.7 (− 0.6 to 5.8)	0.09 (− 0.08 to 0.28)	61.0 (42.1–86.1)	3.4 (0–6.6)	0.10 (− 0.53 to 0.78)
India	491.9 (438.6–548.8)	8.7 (5.5–12.1)*	0.28 (0.10–0.46)*	46.0 (31.8–65.3)	10.2 (6.9–13.8)*	0.32 (− 0.32 to 1.00)
Indonesia	534.0 (474.6–602.9)	4.2 (0.3–8.2)*	0.12 (− 0.08 to 0.32)	52.4 (36.0–73.0)	4.3 (0.2–8.4)*	0.11 (− 0.52 to 0.78)
Italy	710.8 (648.1–772.1)	0.8 (− 5.1 to 6.5)	0.13 (− 0.02 to 0.30)	66.6 (47.0–91.8)	0.4 (− 6.0 to 6.3)	0.12 (− 0.48 to 0.78)
Japan	454.7 (416.0–490.4)	− 3.7 (− 8.4 to 0.5)	− 0.15 (− 0.30 to 0.02)	43.6 (30.8–61.5)	− 4.0 (− 8.9 to 0.9)	− 0.17 (− 0.78 to 0.50)
Mexico	587.8 (523.4–652.2)	6.9 (3.9–10.4)*	0.22 (0.05–0.40)*	55.2 (37.7–77.7)	7.2 (3.9–11.0)*	0.20 (− 0.44 to 0.88)
Republic of Korea	533.3 (480.6–594.2)	3.2 (− 3.3 to 9.8)	0.25 (0.07–0.45)*	51.8 (36.2–73.6)	− 0.7 (− 6.9 to 6.2)	0.11 (− 0.51 to 0.77)
Russian Federation	650.1 (575.9–727.2)	6.0 (2.7–9.3)*	0.26 (0.06–0.48)*	61.0 (42.8–84.5)	4.3 (0.2–7.6)*	0.19 (− 0.42 to 0.84)
Saudi Arabia	715.3 (626.1–811.7)	5.5 (− 1.2 to 12.4)	0.21 (− 0.01 to 0.44)	66.5 (45.8–93.6)	5.5 (− 2.7 to 12.7)	0.20 (− 0.43 to 0.86)
South Africa	616.2 (538.7–695.2)	4.5 (1.8–7.3)*	0.19 (− 0.04 to 0.42)	60.0 (40.9–84.4)	3.9 (0.6–6.8)*	0.15 (− 0.51 to 0.86)
Turkey	776.4 (675.6–866.4)	0.4 (− 5.1 to 6.7)	0.14 (− 0.06 to 0.34)	73.0 (51.8–102.2)	− 0.6 (− 7.1 to 5.4)	0.09 (− 0.52 to 0.76)
United Kingdom	471.0 (413.6–528.7)	1.0 (− 2.4 to 4.3)	0.03 (− 0.18 to 0.24)	44.1 (30.5–63.0)	0.9 (− 2.4 to 4.2)	0.03 (− 0.63 to 0.72)
United States of America	864.3 (789.9–937.3)	0.5 (− 5.6 to 7.0)	− 0.04 (− 0.19 to 0.13)	80.4 (56.4–111.0)	0.9 (− 5.1 to 7.5)	− 0.02 (− 0.62 to 0.64)

*Indicates a significant increase or decrease. EAPC values are presented with two decimal places for greater clarity due to their relatively small magnitude

*YLDs* years lived with disability, *UI* uncertainty interval, *ASR* age-standardized rate, *EAPC* estimated annual percentage change

### Causes of HF

**Fig. 1:**
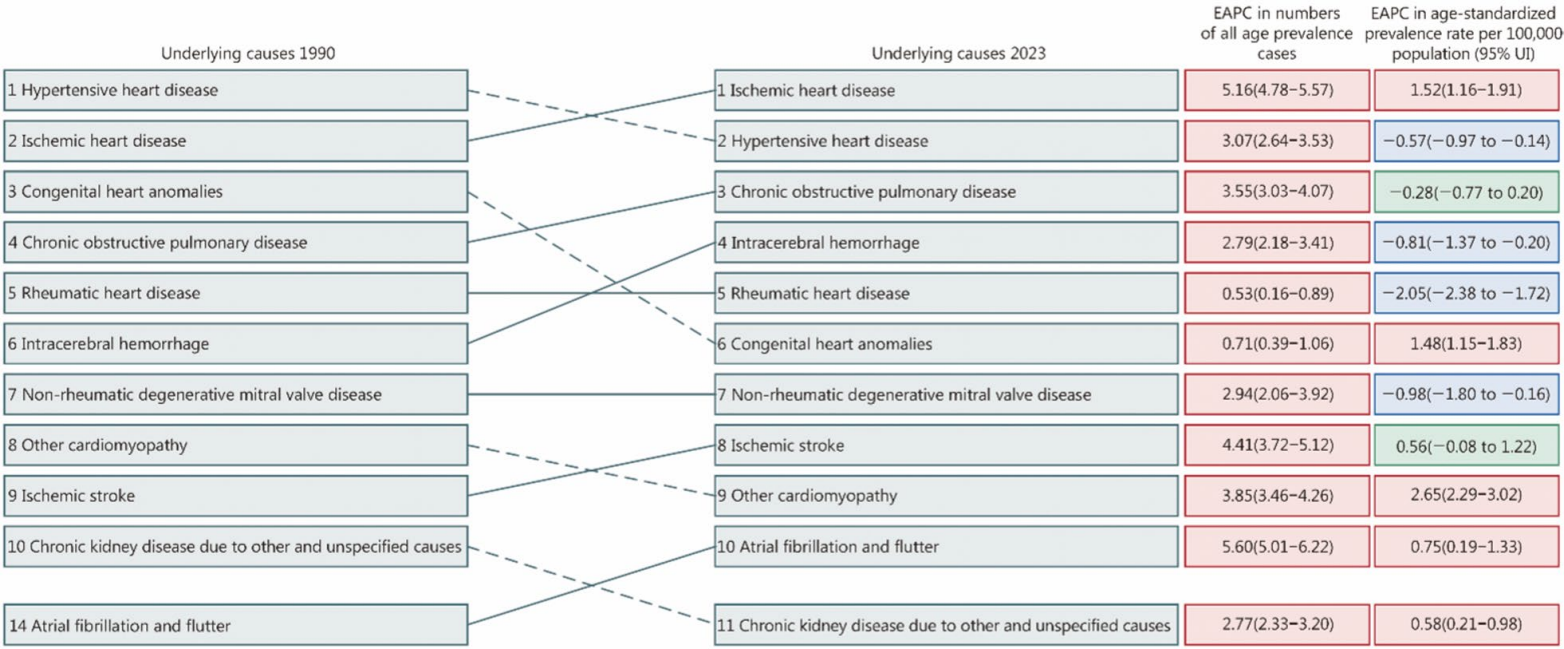
Heart failure due to the top 10 underlying causes rankings by absolute number of prevalence cases in both sexes in China, 1990–2023. Rankings represent the absolute number of prevalence cases. Heart failure due to underlying causes was ranked in 2023, with lines connecting to their rank in 1990. The colors refer to the EAPC in underlying causes from 1990 to 2023: red is an increase in EAPC, green is no change, and blue is a decrease. EAPC estimated annual percent change

In 2023, IHD emerged as the primary cause of HF for both sexes, exhibiting a significant increase in all-age prevalence numbers, ASPR, as well as the proportions of IHD among all underlying causes (Fig. 1; Additional file 1: Tables S5–S9). HHD declined to become the second leading cause, while COPD and intracerebral hemorrhage ascended to the third and fourth place. Congenital heart anomalies declined to become the sixth cause, with the ranks of rheumatic heart disease remaining unchanged from 1990 to 2023 (Fig. 1). The collective proportion of the leading 7 causes accounted for 90% of total HF cases in 2023, underscoring their significant contribution to the overall burden of HF (Additional file 1: Table S10). A significant increase in the ASPR of HF attributed to IHD, congenital heart anomalies, and other cardiomyopathies was observed during the study period (Fig. 1). Conversely, significant decreases were noted in the ASPR associated with HHD, intracerebral hemorrhage, RHD, and non-rheumatic degenerative mitral valve disease. The changes in the ranks of prevalence cases and ASPR for other causes were also shown in Fig. 1. In 2023, the YLDs attributed to HF accounted for 11.90% of the YLDs for CVDs, 2.77% of the YLDs for chronic respiratory diseases, and 0.79% of the total YLDs from all causes of morbidity in China (Table 2).

**Table 2:** Estimated percentage of YLDs rate from heart failure in YLDs rate from all level 2 causes and all-cause in GBD, China, and its provinces in 2023 [% (95% UI)]

Location	CVD	Chronic respiratory diseases	Other non-communicable diseases	Diabetes and kidney diseases	Digestive diseases	Substance use disorders	Neglected tropical diseases and malaria	All causes
China	11.90 (9.69–14.27)	2.77 (1.90–3.69)	0.33 (0.24–0.43)	0.34 (0.25–0.48)	0.26 (0.18–0.36)	0.01 (0.01–0.02)	0	0.79 (0.66–0.92)
Anhui	12.72 (10.30–15.36)	2.81 (1.80–3.93)	0.37 (0.27–0.52)	0.27 (0.18–0.38)	0.17 (0.11–0.24)	0.01 (0.01–0.01)	0	0.91 (0.76–1.09)
Beijing	12.50 (9.92–15.04)	2.06 (1.29–3.06)	0.26 (0.19–0.34)	0.39 (0.26–0.56)	0.31 (0.21–0.43)	0.01 (0.01–0.01)	0	0.76 (0.62–0.89)
Chongqing	12.07 (9.82–14.76)	4.61 (3.12–5.97)	0.27 (0.19–0.36)	0.36 (0.24–0.54)	0.29 (0.19–0.42)	0.01 (0.01–0.02)	0	0.84 (0.70–1.00)
Fujian	13.95 (11.40–16.87)	2.79 (1.98–3.75)	0.42 (0.31–0.56)	0.37 (0.26–0.53)	0.33 (0.23–0.47)	0.01 (0.01–0.01)	0	0.70 (0.59–0.82)
Gansu	11.21 (8.97–13.79)	3.34 (2.23–4.48)	0.32 (0.23–0.43)	0.35 (0.24–0.50)	0.25 (0.17–0.35)	0.01 (0.01–0.01)	0	0.75 (0.62–0.88)
Guangdong	13.61 (11.00–16.21)	2.35 (1.54–3.27)	0.41 (0.29–0.55)	0.36 (0.26–0.49)	0.27 (0.18–0.37)	0.01 (0.01–0.02)	0	0.61 (0.51–0.69)
Guangxi	12.50 (10.17–14.91)	2.80 (1.92–3.84)	0.34 (0.25–0.45)	0.44 (0.28–0.64)	0.32 (0.22–0.44)	0.03 (0.02–0.04)	0	0.82 (0.68–0.95)
Guizhou	10.14 (8.17–12.31)	3.33 (2.35–4.35)	0.34 (0.24–0.46)	0.40 (0.28–0.57)	0.39 (0.27–0.56)	0.02 (0.02–0.03)	0	0.64 (0.54–0.76)
Hainan	11.86 (9.72–13.99)	2.72 (1.87–3.66)	0.37 (0.26–0.51)	0.27 (0.19–0.38)	0.36 (0.25–0.51)	0.03 (0.02–0.04)	0	0.69 (0.58–0.80)
Hebei	10.96 (8.74–13.42)	1.23 (0.78–1.98)	0.38 (0.26–0.53)	0.25 (0.17–0.34)	0.14 (0.09–0.20)	0.02 (0.01–0.03)	0	0.80 (0.67–0.95)
Heilongjiang	10.16 (8.13–12.11)	2.25 (1.46–3.35)	0.20 (0.14–0.26)	0.18 (0.12–0.27)	0.23 (0.15–0.32)	0.01 (0.01–0.01)	0	0.83 (0.67–0.97)
Henan	11.68 (9.28–14.12)	2.03 (1.31–2.99)	0.45 (0.31–0.62)	0.31 (0.21–0.43)	0.16 (0.10–0.22)	0.01 (0.01–0.01)	0	0.84 (0.69–0.99)
Hong Kong SAR	14.38 (11.49–17.38)	2.28 (1.45–3.37)	0.17 (0.12–0.25)	1.20 (0.80–1.80)	0.41 (0.28–0.59)	0.05 (0.03–0.07)	0	0.80 (0.65–0.95)
Hubei	12.67 (10.08–15.44)	2.54 (1.63–3.65)	0.30 (0.21–0.40)	0.37 (0.25–0.53)	0.21 (0.14–0.31)	0.01 (0.01–0.01)	0	0.81 (0.68–0.97)
Hunan	13.00 (10.60–15.72)	2.49 (1.63–3.56)	0.32 (0.23–0.44)	0.45 (0.30–0.64)	0.22 (0.14–0.31)	0.01 (0.01–0.02)	0	0.88 (0.72–1.04)
Inner Mongolia	10.19 (8.03–12.38)	1.61 (1.04–2.35)	0.25 (0.19–0.33)	0.20 (0.14–0.30)	0.20 (0.13–0.28)	0.01 (0.00–0.01)	0	0.73 (0.60–0.87)
Jiangsu	12.79 (10.32–15.43)	3.84 (2.63–5.06)	0.32 (0.23–0.44)	0.35 (0.23–0.50)	0.29 (0.20–0.40)	0.01 (0.01–0.01)	0	0.91 (0.76–1.06)
Jiangxi	11.91 (9.72–14.45)	2.78 (1.92–3.73)	0.39 (0.28–0.54)	0.46 (0.33–0.67)	0.31 (0.21–0.43)	0.01 (0.01–0.02)	0	0.74 (0.61–0.85)
Jilin	10.47 (8.47–12.83)	1.57 (0.96–2.46)	0.23 (0.17–0.31)	0.25 (0.17–0.36)	0.26 (0.18–0.37)	0.01 (0.01–0.01)	0	0.87 (0.70–1.02)
Liaoning	10.39 (8.25–12.69)	1.97 (1.24–3.02)	0.23 (0.17–0.30)	0.26 (0.17–0.39)	0.28 (0.18–0.39)	0.01 (0.01–0.02)	0	0.89 (0.72–1.07)
Macao SAR	11.19 (8.98–13.32)	4.14 (3.08–5.25)	0.30 (0.22–0.40)	1.27 (0.86–1.80)	0.34 (0.23–0.46)	0.09 (0.06–0.12)	0	0.71 (0.60–0.82)
Ningxia	10.61 (8.65–12.88)	2.26 (1.49–3.23)	0.37 (0.27–0.51)	0.30 (0.20–0.42)	0.26 (0.17–0.35)	0.01 (0.01–0.01)	0	0.64 (0.52–0.75)
Qinghai	9.27 (7.61–11.33)	2.37 (1.71–3.13)	0.32 (0.23–0.44)	0.33 (0.23–0.46)	0.29 (0.20–0.40)	0.01 (0.01–0.02)	0	0.60 (0.50–0.70)
Shaanxi	10.79 (8.72–13.20)	2.20 (1.39–3.23)	0.32 (0.23–0.44)	0.33 (0.23–0.49)	0.22 (0.14–0.31)	0.01 (0.01–0.02)	0	0.79 (0.65–0.93)
Shandong	11.75 (9.41–14.50)	2.67 (1.69–3.72)	0.35 (0.26–0.47)	0.27 (0.19–0.41)	0.18 (0.12–0.25)	0.01 (0.01–0.01)	0	0.89 (0.73–1.05)
Shanghai	14.81 (11.89–18.05)	3.73 (2.63–4.87)	0.24 (0.18–0.31)	0.57 (0.38–0.81)	0.51 (0.34–0.69)	0.02 (0.01–0.02)	0	0.85 (0.70–0.99)
Shanxi	10.51 (8.31–12.77)	2.50 (1.60–3.73)	0.34 (0.25–0.47)	0.24 (0.17–0.35)	0.19 (0.13–0.26)	0.01 (0.01–0.01)	0	0.81 (0.66–0.95)
Sichuan	11.62 (9.28–14.06)	4.27 (3.10–5.65)	0.22 (0.16–0.31)	0.42 (0.29–0.62)	0.37 (0.25–0.52)	0.02 (0.02–0.03)	0	0.78 (0.64–0.90)
Tianjin	10.95 (8.64–13.31)	1.67 (1.09–2.56)	0.30 (0.22–0.40)	0.26 (0.18–0.38)	0.20 (0.13–0.29)	0.01 (0.01–0.02)	0	0.85 (0.69–0.99)
Xizang	10.27 (8.45–12.30)	1.19 (0.75–1.81)	0.44 (0.30–0.59)	0.47 (0.35–0.63)	0.49 (0.34–0.67)	0.00 (0.00–0.01)	0	0.48 (0.40–0.55)
Xinjiang	11.62 (9.57–13.78)	1.95 (1.35–2.60)	0.37 (0.25–0.54)	0.30 (0.22–0.40)	0.16 (0.11–0.22)	0.03 (0.02–0.04)	0	0.67 (0.57–0.79)
Yunnan	10.24 (8.11–12.36)	3.60 (2.52–4.52)	0.26 (0.19–0.33)	0.36 (0.25–0.53)	0.31 (0.21–0.43)	0.02 (0.01–0.02)	0	0.61 (0.51–0.72)
Zhejiang	14.43 (11.54–17.62)	3.45 (2.27–4.63)	0.29 (0.22–0.38)	0.46 (0.31–0.69)	0.34 (0.23–0.49)	0.01 (0.01–0.02)	0	0.77 (0.64–0.91)

Percentage values are presented with two decimal places for greater clarity due to their relatively small magnitude. *UI* uncertainty interval, *YLDs* years lived with disability, *SAR* Special Administrative Regions, *GBD* Global Burden of Diseases, *CVD* cardiovascular diseases

### Age and sex differences

**Fig. 2:**
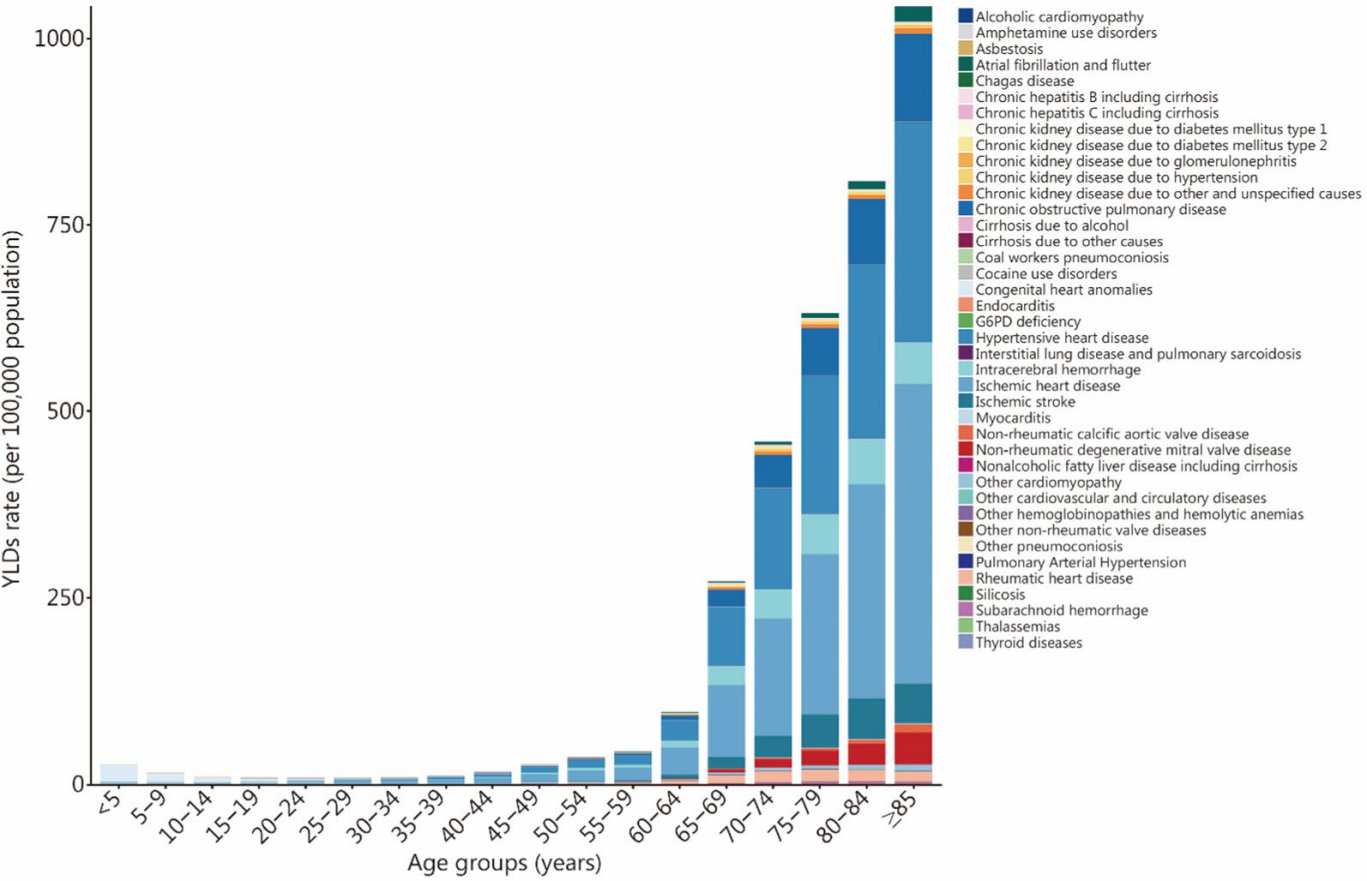
YLDs rates of heart failure due to 40 underlying causes by age group, 2023. YLDs years lived with disability

In 2023, the YLDs rate of HF in adults increased with age in both sexes, with the primary cause varying across different age groups. In children and adolescents, congenital heart anomalies ranked as the leading cause, followed by other cardiomyopathies, collectively constituting over 75% of HF burden within this age group. In individuals aged 15–49 years, IHD and HHD rose in rank to become the two leading causes. In individuals aged 50 and above, IHD, HHD, and COPD emerged as the top three causes (Fig. 2). In 2023, the overall prevalence and YLDs rate of HF in males was higher than that in females, with sex disparities varying across different causes (Additional file 1: Figs. S1, S2).

### Regional disparities

**Fig. 3:**
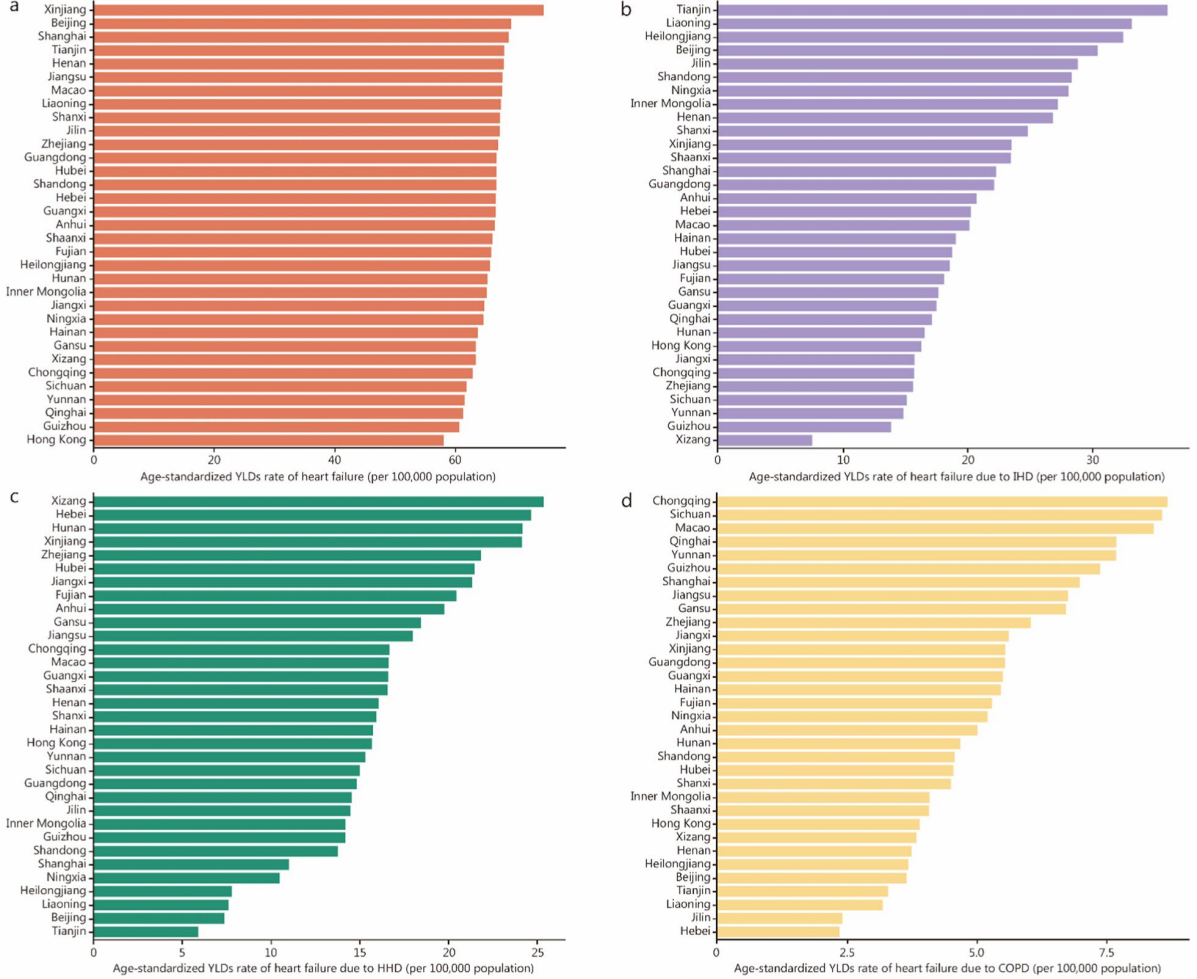
Age-standardized YLDs rate of overall heart failure (**a**), heart failure due to ischemic heart disease (**b**), hypertensive heart disease (**c**), and chronic obstructive pulmonary disease (**d**) across provinces in 2023. YLDs years lived with disability, IHD ischemic heart disease, HHD hypertensive heart disease, COPD chronic obstructive pulmonary disease

In 2023, marked heterogeneity was observed in the burden of HF across provinces. The highest age-standardized YLDs rates of HF were seen in Xinjiang, nearly 1.3 times the lowest province, Hong Kong SAR (Fig. 3a). All-age prevalence and YLDs rates across provinces by severity are shown in Additional file 1: Figs. S3, S4. Distinct spatial patterns were observed in the provincial distribution of age-standardized YLDs for HF attributed to the 3 leading causes, namely IHD, HHD, and COPD. A marked north–south disparity was observed in the age-standardized YLDs rate for HF caused by IHD, which was particularly higher in northern provinces such as Tianjin, Liaoning, and Heilongjiang, more than fourfold that of the province with the lowest rate, Xizang (Fig. 3b). Xizang, Hebei, Hunan, and Xinjiang had the highest burden of HF due to HHD **(**Fig. 3c). Western provinces, especially Chongqing and Sichuan, had the highest age-standardized YLDs rates for HF due to COPD, and northern provinces had the lowest rates (Fig. 3d). Rankings of underlying causes varied from the province, but IHD, HHD, and COPD remained the top three leading causes by age-standardized prevalence and YLDs rate in most provinces, which contributed to more than 60.00% of the total burdens of HF in those provinces (Additional file 1: Figs. S5, S6). The proportion of CVD YLDs attributable to HF within the CVD YLDs rate exceeded 14.30% in some eastern provinces, including Shanghai, Zhejiang, and Hong Kong SAR (Table 2).

Our quadrant analysis revealed that six of the 11 provinces with the highest burden of disability of HF in 2023 had also experienced the greatest increase between 1990 and 2023. These provinces included Liaoning, Heilongjiang, and Jilin in northeastern China, Sichuan and Chongqing in the southwest region, and Anhui in the central part. In contrast, western border provinces (Xizang, Xinjiang) and southeastern coastal provinces (Guangdong, Hainan, Fujian, and Macao SAR) exhibited the lowest combined YLDs burdens, with the smallest increase in YLDs burden from 1990 to 2023. The remaining provinces were classified into other quadrants (Additional file 1: Fig. S7).

## Discussion

This study, using data from GBD, systematically depicted the prevalence, YLDs, and underlying causes of HF from 1990 to 2023 in China on both national and subnational scales. Our findings demonstrated that both the prevalence and YLDs rates of HF in China increased consistently in the past three decades. HF in China maintained a heavier burden compared to the global average level in 2023. IHD, HHD, and COPD were the top three causes of HF in China in 2023. The burden of HF, as indicated by YLDs rates, exhibited great heterogeneity across provinces. Distinct geographical patterns hold the potential for informing resource allocation and spatially targeted strategy. Given the substantial and rapid aging of the population, HF persists as an escalating public health concern in China, warranting heightened attention and resources.

Although the ASPRs and YLDs rates of China exceeded the global average slightly, the absolute number of prevalence cases represented more than one-quarter of the global total. In 2023, 14.3 million individuals were living with heart failure in China. This estimate aligns closely with findings from a study utilizing data from the China Health Insurance Database, which reported a crude prevalence rate of 1.18% among individuals aged 25 years and above in 2017, equating to an estimated 12.1 million patients [7]. The approximate estimation results corroborate the precision and reliability of the estimates derived from the GBD 2023 dataset. These data underscore the immense burden of heart failure that China is grappling with, emphasizing the critical need for targeted interventions and robust healthcare strategies to address this challenge.

We have observed a consistent increase in age-standardized HF prevalence rates and YLDs rates over the past three decades. This upward trend might be attributed to multiple factors. Firstly, the rising prevalence of risk factors such as hypertension [21], diabetes [22], obesity [23], and dyslipidemia [24], among others, between 1990 and 2023, has resulted in a surge of cardiovascular diseases like coronary heart disease [9], arrhythmias [25], and valvular heart diseases [26]. Consequently, a portion of these diseases inevitably progressed to HF [9]. Secondly, advancements in medication and device therapies in recent decades have substantially extended the life expectancy of patients with HF. Since the 1990s, HF treatment has evolved from short-term hemodynamic improvement to long-term reparative strategies aimed at altering the biological nature of the failing heart. This transition has involved moving from inotropic, diuretic, and vasodilator drugs to neurohormonal antagonists. Notably, the advent of renin-angiotensin-aldosterone system inhibitors [27–29], mineralocorticoid receptor antagonists [30], beta blockers, and sodium-glucose cotransporter-2 inhibitors (SGLT2i) [31] is associated with a reduction in the mortality rate of HF with decreased ejection fraction. Furthermore, guideline-directed device therapies such as implantable cardioverter-defibrillators and cardiac resynchronization therapies have rapidly advanced, been widely adopted in clinical practice, and significantly improved the prognosis of HF patients [32]. Thirdly, continuous promotion of standardized HF care management within the healthcare system [33] along with increased health insurance coverage, has also contributed to better HF prognosis. Anticipated is a continued increase in the prevalence of heart failure cases in the future, driven by the ongoing efforts of the healthcare system and the evolution of treatment approaches.

There have been minor shifts in the ranking of ASPRs by causes over the past three decades. CVD has consistently remained the predominant cause of HF in China, accounting for 80.1% in 1990 and rising to 84.8% in 2023 of all HF cases. Specifically, IHD, HHD, and intracerebral hemorrhage emerged as the top three leading causes of HF attributed to CVD in 2023. The prevalence rates for HF attributed to atherosclerotic CVD, particularly IHD, have witnessed a significant and rapid increase over the past three decades. This surge can be largely attributed to long-standing lifestyle and metabolic risk factors, and exposure to air pollution and climate variations [34]. Despite a decrease in prevalence rates of HF associated with HHD, hypertension remains prevalent in China, with suboptimal blood pressure control among hypertensive individuals when compared to high-income countries [35]. Noteworthy progress has been made in reducing HF prevalence rates due to RHD over the study period, attributed to rapid socioeconomic developments with substantial improvements in hygiene and living conditions that have led to a decline in RHD incidence [36]. The premature mortality burden of congenital heart anomalies has decreased following the nationwide policy implementation of newborn screening since 2009 [37]. While there has been a substantial decrease in prevalence rates of HF due to COPD from 1990 to 2023, COPD remains a significant public health issue in China, with a higher prevalence among individuals aged 40 years and above compared to global estimates [38]. High smoking rates among Chinese males, coupled with low awareness of COPD and inadequate spirometry testing in the general population, contribute to this concern [39]. Risk control programs targeting reductions in ambient PM2.5 pollution and smoking have been initiated by the Chinese government, but progress has been made only in recent years [39–41]. These identified causes can serve as a foundation for guiding HF prevention and control priorities, influencing intervention strategies to combat the burden of heart failure effectively.

Significant geographical disparities in age-standardized YLDs rates and their leading causes persist across provinces in China. Provinces with a high burden in 2023 and substantial increases over the past three decades, such as Liaoning, Jilin, Heilongjiang, Sichuan, Chongqing, and Anhui, face greater challenges in addressing HF and should be prioritized by health policymakers. The higher relative burden of heart failure, indicated by the proportion of YLDs attributed to heart failure within the overall YLDs for CVDs, in eastern provinces such as Shanghai, Zhejiang, and Hong Kong SAR, underscores the need for improved treatment and management of CVDs in these regions to delay the progression to heart failure. In line with our results, similar research revealed regional differences in HF in China. A nationwide representative survey across China in 2000 reported that the prevalence of HF was higher in the northern than in southern China [17]. Findings from the China Health Insurance Database also showed that a relatively higher standard prevalence of HF in 2017 was observed in Liaoning, Zhejiang, and Qinghai provinces than in Inner Mongolia, Chongqing, and Hainan provinces [7]. Factors contributing to regional variations included socioeconomic and demographic disparities [42], differences in the prevalence status of HF risk factors [9, 43, 44], disparities in levels of prevention and control of these risk factors, and variations in public health policy and healthcare service capability [45]. Additionally, the availability and accessibility of medical resources, along with gaps in the quality of treatment, care, and patient management [46–48], further exacerbated these disparities. This highlights the urgent need for region-specific strategies for HF prevention, treatment, and management, and calls for increased investment in medical resources in certain provinces. Strengthening guideline-directed medical therapies (GDMT) is essential for treatment, improving patient prognosis, and preventing readmission. However, disparities in the quality of HF care and adherence to guidelines remained across regions and hospital grades [33, 49]. Hospitals in eastern China showed better composite performances of quality of HF care than hospitals in other regions, including left ventricular ejection fraction assessment during hospitalization, the usage of beta-blockers, angiotensin-converting enzyme inhibitors, and angiotensin receptor blockers, and follow-up appointments at discharge [50]. HF patients discharged from secondary hospitals showed higher all-cause and CVD mortality than those from tertiary hospitals [49]. These variations may be linked to inequalities in medical resource allocation and differences in the knowledge and treatment capabilities of local healthcare providers at various hospital levels. In response, China launched the HF Center Program in 2017, establishing standardized HF centers in secondary and tertiary hospitals across all 31 provinces. This initiative aims to establish a comprehensive system for the standardized management of HF, promoting GDMT, and minimizing regional disparities [51]. The establishment of HF achieved notable progress, for example, the usage rate of SGLT2i at discharge was significantly higher in accredited HF centers compared with non-accredited HF centers (47.1% vs. 35.8%) [52]. However, the HF clinical practice is still faced with a low usage rate of GDMT and gaps with targets recommended by HF guidelines, calling for more investment in standard HF management. Special efforts are also needed to improve the generalizability and accessibility of medication and device therapies and to strengthen technical training for clinicians in economically disadvantaged regions and provinces.

Our findings have important implications for both national and subnational governments, guiding the formulation of region-specific public health and clinical policies or programs targeting population-specific risks and underlying causes, to prevent and manage HF. Given the aging population and the increasing prevalence of non-communicable diseases, our study suggests intensified efforts are needed to control the prevailing atherosclerotic CVD and COPD to mitigate the HF challenges. Identifying and controlling modifiable risk factors and preventing the onset of primary causes should be priorities in HF prevention. Cost-effective monitoring, health education, and promotion initiatives focusing on adherence to healthy lifestyles and raising public awareness of modifiable risk factors are also essential, particularly for high-risk populations [53]. For individuals with cardiovascular diseases, targeted and optimal treatments are imperative to prevent or delay the progression of HF. Early cost-effective screening and treatment for those at pre-HF stages (stage B HF) are recommended by existing guidelines and are crucial to improving prognosis [54]. Given the variation in leading HF causes across different provinces, each province should assess and manage its specific leading causes and implement targeted interventions. For example, provinces with high HF burdens (e.g., Liaoning, Heilongjiang, and Jilin) should prioritize implementing intensified IHD screening programs and early therapeutic interventions. Similarly, targeted HHD screening initiatives are recommended for Sichuan, Chongqing, and Anhui provinces. Furthermore, Sichuan and Chongqing provinces require additional emphasis on enhancing COPD screening, surveillance, and management. Furthermore, international and nationwide experience, such as more strike tobacco control legislation in Japan [55], air pollution action in China [56], and application of artificial intelligence to HF prediction [57], could be used to improve HF prevention and management.

To our knowledge, this study presents the first comprehensive analysis of spatiotemporal variations in HF burden across provincial-level regions in China and provides suggestions on regional targeted strategies to promote health equity in HF. Our findings could provide evidence for the local governments to take measures to mitigate HF burden, and also, provide a reference for HF prevention and control in some low- and middle-income countries. Despite the robustness of the methodology and estimates in the GBD 2023 study, several limitations should be noted. First, the GBD 2023 estimated the models of HF mainly by reviewing all available data at the national and sub-national levels, yet the lack of relevant data in some provinces might result in biased estimations and underestimations due to low diagnostic rates in some less-developed regions. Second, the GBD assumed that each HF patient only has one etiology, although comorbidity or combined causes are possible. This assumption was made to enhance the utility of the estimates and to identify the most common and clinically relevant contributors. However, the potential conflict between the GBD assumption of “single cause” and actual co-existing causes might underestimate the impact of complex cases with multiple comorbidities on HF burden. Though many diseases can lead to HF, a detailed analysis of all underlying causes may not be feasible, and some results may remain inconclusive. However, GBD categorized etiologies based on the pathophysiologic or causal mechanism and used standardized case definitions, providing public health pathways for prevention [58]. Finally, this study was subjected to all the inherent limitations of the GBD studies [12].

## Conclusions

In conclusion, HF continues to pose a significant public health concern in China. Policymakers should prioritize the development of targeted and region-specific public health strategies, including those that focus on early screening for high-risk populations, effective surveillance and control of HF’s main causes, and the optimization of healthcare resources. Furthermore, nationwide enhancement of the standard diagnosis, treatment, and management of HF patients based on GDMT could reduce the geographical disparities in the burden of HF.

## Supplementary Information

**Additional file 1.** **Table S1** Severity distribution, details on the severity levels for heart failure in GBD 2023, and the associated DW with that severity. **Table S2** List of International Classification of Diseases codes mapped to underlying causes of heart failure for GBD 2023. **Table S3 **Number, rate, and age-standardized prevalence rate of heart failure, and percentage change from 1990 to 2023 by severity and sex. **Table S4** Number, rate, and age-standardized YLDs rate of heart failure, and percentage change from 1990 to 2023 by severity and sex. **Table S5** Number (in thousands) and proportion of overall heart failure by cause in China in 1990 and 2023 [number in thousands (%)]. **Table S6** Number (in thousands) and proportion of treated heart failure by cause in China in 1990 and 2023 [number in thousands (%)]. **Table S7** Number (in thousands) and proportion of mild heart failure by cause in China in 1990 and 2023 [number in thousands (%)]. **Table S8** Number (in thousands) and proportion of moderate heart failure by cause in China in 1990 and 2023 [number in thousands (%)]. **Table S9** Number (in thousands) and proportion of severe heart failure by cause in China in 1990 and 2023 [number in thousands (%)]. **Table S10** Top 10 causes of heart failure by all-age prevalence rate and proportion in all causes in 2023 and EAPC in prevalence rate in China, 1990 – 2023. **Fig. S1** Age-standardized prevalence of heart failure due to 40 causes by sex, 2023. **Fig. S2** Heart failure due to underlying causes rankings by absolute number of YLDs in males (a) and females (b) in China, 1990 – 2023. YLDs years lived with disability. **Fig. S3** Prevalence rate of heart failure (per 100,000 population) in China, 2023. **Fig. S4** YLDs rate of heart failure (per 100,000 population) in China, 2023. YLDs years lived with disability. **Fig. S5** Age-standardized prevalence rate of heart failure due to 40 causes in China, 2023. **Fig. S6 **Age-standardized YLDs rate of heart failure due to 40 causes in China, 2023. YLDs years lived with disability. **Fig. S7** All-age YLDs rates per 100,000 population in 2023 and estimated annual percentage change in YLDs rates per 100,000 population in 1990 – 2023 due to heart failure for all ages and both sexes in China. YLDs years lived with disability.

## Data Availability

The datasets analyzed during the current study are available on the Global Health Data Exchange GBD 2023 website.

## Abbreviations

ASPR: Age-standardized prevalence rate
COPD: Chronic obstructive pulmonary disease
CVD: Cardiovascular disease
EAPC: Estimated annual percentage change
GATHER: Guidelines for Accurate and Transparent Health Estimates Reporting
GBD: Global Burden of Diseases
GDMT: Guideline-directed medical therapies
HF: Heart failure
HHD: Hypertensive heart disease
ICD: International Classification of Diseases
IHD: Ischemic heart disease
SAR: Special administrative region
SGLT2i: Sodium-glucose cotransporter-2 inhibitor
UI: Uncertainty interval
YLD: Year lived with disability
