# Supplementary material for: The evolving burden of heart failure in China: a 34-year subnational analysis of trends and causes from the Global Burden of Disease Study 2023
Source: Mil Med Res. 2025 Oct 9;12:65. doi: 10.1186/s40779-025-00650-y (PMC12509353; doi:10.1186/s40779-025-00650-y)
Supplement: Supplementary file 1 — Additional file 1. Table S1 Severity distribution, details on the severity levels for heart failure in GBD 2023, and the associated DW with that severity. Table S2 List of International Classification of Diseases codes mapped to underlying causes of heart failure for GBD 2023. Table S3 Number, rate, and age-standardized prevalence rate of heart failure, and percentage change from 1990 to 2023 by severity and sex. Table S4 Number, rate, and age-standardized YLDs rate of heart failure, and percentage change from 1990 to 2023 by severity and sex. Table S5 Number (in thousands) and proportion of overall heart failure by cause in China in 1990 and 2023 [number in thousands (%)]. Table S6 Number (in thousands) and proportion of treated heart failure by cause in China in 1990 and 2023 [number in thousands (%)]. Table S7 Number (in thousands) and proportion of mild heart failure by cause in China in 1990 and 2023 [number in thousands (%)]. Table S8 Number (in thousands) and proportion of moderate heart failure by cause in China in 1990 and 2023 [number in thousands (%)]. Table S9 Number (in thousands) and proportion of severe heart failure by cause in China in 1990 and 2023 [number in thousands (%)]. Table S10 Top 10 causes of heart failure by all-age prevalence rate and proportion in all causes in 2023 and EAPC in prevalence rate in China, 1990 – 2023. Fig. S1 Age-standardized prevalence of heart failure due to 40 causes by sex, 2023. Fig. S2 Heart failure due to underlying causes rankings by absolute number of YLDs in males (a) and females (b) in China, 1990 – 2023. YLDs years lived with disability. Fig. S3 Prevalence rate of heart failure (per 100,000 population) in China, 2023. Fig. S4 YLDs rate of heart failure (per 100,000 population) in China, 2023. YLDs years lived with disability. Fig. S5 Age-standardized prevalence rate of heart failure due to 40 causes in China, 2023. Fig. S6 Age-standardized YLDs rate of heart failure due to 40 causes in China, 2023. YLDs years [file 40779_2025_650_MOESM1_ESM.pdf]

Table S1 Severity distribution, details on the severity levels for heart failure in GBD 2023, and the associated DW with that severity

Severity level	Lay description	DW (95% CI)
Controlled, medically managed	Has been diagnosed with clinical heart failure, a chronic disease that requires medication every day and causes some worry but minimal interference with daily activities?	0.049 (0.031 – 0.072)
Mild	Is short of breath and easily tired with moderate physical activity, such as walking uphill or more than a quarter-mile on level ground? The person feels comfortable at rest or during activities requiring less effort	0.041 (0.026 – 0.062)
Moderate	Is short of breath and easily tired with minimal physical activity, such as walking only a short distance? The person feels comfortable at rest but avoids moderate activity	0.072 (0.047 – 0.103)
Severe	Is short of breath and easily tired with minimal physical activity, such as walking only a short distance? The person feels comfortable at rest but avoids moderate activity	0.179 (0.122 – 0.251)

GBD Global Burden of Diseases, *DW* disability weight, *CI* confidential interval

Table S2 List of International Classification of Diseases codes mapped to underlying causes of heart failure for GBD 2023

Level	Cause	ICD-10	ICD-10 used in hospital/Claims analyses	ICD-9	ICD-9 used in hospital/Claims analyses
**2**	**Neglected tropical diseases and malaria**	-	-	-	-
3	Chagas disease	B57 – B57.5, K93.1	-	086 – 086.2, 425.6	-
**2**	**Cardiovascular diseases**	-	-	-	-
3	Rheumatic heart disease	I01 – I01.9, I02.0, I05 – I09.9	I01 – I09.9	391 – 391.9, 392.0, 393 –398.99	391 – 398.99
3	Ischemic heart disease	I20 – I21.6, I21.9 – I25.9, Z82.4 – Z82.49	I20 – I25.9	410 – 414.9, V17.3	410 – 414.9
3	Stroke	G45 – G46.8, I60 – I62, I62.9 – I64, I64.1, I65 – I69.998, Z82.3	I60 – I69.4	430 – 439.6, V12.54, V17.1	430 – 437.9
4	Ischemic stroke	G45 – G46.8, I63 – I63.9, I65 – I66.9, I67.2 – I67.848, I69.3 – I69.4	I63 – I63.9	433 – 435.9, 437.0 – 437.2, 437.4 – 437.9	434 – 434.91
4	Intracerebral hemorrhage	I61 – I62, I62.9, I69.0 – I69.298	I61 – I62.9	431, 431.1 – 432.9	431, 431.1 – 432.9
4	Subarachnoid hemorrhage	I60 – I60.9, I67.0 – I67.1	I60 – I60.9, I67.0 – I67.1	430 – 430.9, 431.0, 437.3	430 – 430.9, 431.0, 437.3
3	Hypertensive heart disease	I11 – I11.2, I11.9	-	402 – 402.91	
3	Non-rheumatic valvular heart disease	I34 – I37.9	I34 – I37.9	424.0 – 424.3	424.0 – 424.3

Level	Cause	ICD-10	ICD-10 used in hospital/Claims analyses	ICD-9	ICD-9 used in hospital/Claims analyses
4	Non-rheumatic calcific aortic valve disease	-	I35 – I35.9	-	424.1
4	Non-rheumatic degenerative mitral valve disease	-	I34 – I34.9	-	424.0
4	Other non-rheumatic valve diseases	-	I36 – I37.9	-	424.2 – 424.3
3	Cardiomyopathy and myocarditis	B33.2 – B33.20, B33.22 – B33.24, D86.85, I40 – I41.8, I42 – I43.8, I51.4 – I51.6	B33.2 – B33.20, B33.22 – B33.24, I40 – I41.8, I51.4 – I51.6	074.2, 074.23, 422 – 422.99, 425 – 425.5, 425.7 – 425.9, 429.0 – 429.1	074.2, 074.23, 422 – 422.99, 429.0 – 429.1
4	Myocarditis	B33.2 – B33.20, B33.22 – B33.24, D86.85, I40 – I41.8, I51.4 – I51.6	B33.2 – B33.20, B33.22 – B33.24, I40 – I41.8, I51.4 – I51.6	074.2, 074.23, 422 – 422.99, 429.0 – 429.1	074.2, 074.23, 422 – 422.99, 429.0 – 429.1
4	Alcoholic cardiomyopathy	I42.6	-	425.5	416.0
4	Other cardiomyopathy	I42.0 – I42.5, I42.7	-	425.0 – 425.18, 425.3, 425.8 – 425.9	-
3	Pulmonary arterial hypertension	-	I27.0	416.0	-
3	Atrial fibrillation and flutter	I48 – I48.92	I48 – I48.92	427.3 – 427.32	427.3 – 427.32
3	Endocarditis	B33.21, I33 – I33.9, I38 – I38.0, I39 – I39.9	A32.82, B33.21, B37.6, I33 – I33.9, I38 – I39.9	074.22, 421 – 421.9, 424, 424.4 – 424.99	074.22, 112.81 – 115.94, 421 – 421.9, 424, 424.4 – 424.99
3	Other cardiovascular and circulatory diseases	I30 – I32.8, I51 – I51.3, I51.7 – I52.8, I62.0 – I62.1, I72 -I72.9, I77 – I83.93, I86 – I89.0, I89.9, I95.0 – I95.1, I98, I98.8 – I99.9, K75.1	-	074.21, 417 – 417.9, 420 – 420.99, 423 – 423.9, 429, 429.2 - 429.9, 442 – 442.9, 443.21 – 443.29, 447 – 454.9, 456, 456.3 -457, 457.1, 457.8 – 458.1, 459 – 459.9	-

Level	Cause	ICD-10	ICD-10 used in hospital/Claims analyses	ICD-9	ICD-9 used in hospital/Claims analyses
**2**	**Chronic respiratory diseases**	-	-	-	-
3	Chronic obstructive pulmonary disease	J41 – J42.4, J43 – J44.9	J41 – J44.9	491 – 492.9, 496 – 499	491 – 492.9, 496 – 499
3	Pneumoconiosis	J60 – J65.0, J92.0	J60 – J65.0, J92.0	500 – 505.9	500 – 505.9
4	Silicosis	J62 – J62.9	J62 – J62.9	502 – 502.9	502 – 502.9
4	Asbestosis	J61 – J61.0, J92.0	J61 – J61.0, J92.0	501 – 501.9	501 – 501.9
4	Coal workers pneumoconiosis	J60 – J60.0	J60 – J60.0	500 – 500.9	500 – 500.9
4	Other pneumoconiosis	J63 – J65.0	J63 – J65.0	503 – 505.9	503 – 505.9
3	Interstitial lung disease and pulmonary sarcoidosis	D86 – D86.2, D86.9, J84 – J84.9	D86 – D86.2, D86.9, J84 – J84.9	135 – 135.9, 515, 515.9 – 516.9	135 – 135.9, 515 – 516.9
**2**	**Digestive diseases**	-	-	-	-
3	Cirrhosis and other chronic liver diseases	I85 – I85.9, I98.2, K70 – K71, K71.3 – K72, K72.1 – K75, K75.2, K75.4 – K76.2, K76.4 – K77.8, R16 – R18.9, Z52.6, Z94.4	I85 – I85.9, I98.2 – I98.2, K70 – K71, K71.3 – K72, K72.1 – K77.8, R16 – R18.9, Z52.6, Z94.4	456.0 – 456.21, 570 – 572, 572.2 – 573.9, V42.7, V59.6	456.0 – 456.21, 570 – 573.9, V42.7, V59.6
4	Chronic hepatitis B including cirrhosis	-	-	-	-
4	Chronic hepatitis C including cirrhosis	-	-	-	-
4	Cirrhosis due to alcohol	-	-	-	-
4	Nonalcoholic fatty liver disease including cirrhosis	-	K76.0	-	571.9
4	Cirrhosis due to other causes	-	-	-	-

Level	Cause	ICD-10	ICD-10 used in hospital/Claims analyses	ICD-9	ICD-9 used in hospital/Claims analyses
2	Substance use disorders	-	-	-	-
3	Drug use disorders	F11 – F19.99, P96.1, R78.1 – R78.9, Z81.2 – Z81.4	F11 – F15.99	292 – 292.9, 304 – 304.93, 305.1 – 305.93, E850.0 – E850.29, V15.8 – V15.83, V15.85 – V15.86	304.0 – 305.73, E85.00 – E85.029
4	Cocaine use disorders	F14 – F14.99, R78.2	F14 – F14.99	304.20 – 304.23, 305.2 – 305.23, 305.6 – 305.63	304.2 – 304.23, 305.2 – 305.23, 305.6 – 305.63
4	Amphetamine use disorders	F15 – F15.99	F15 – F15.99	304.4 – 304.43, 305.7 – 305.73	304.4 – 304.43, 305.7 – 305.73
**2**	**Diabetes and kidney diseases**	-	-	-	-
3	Chronic kidney disease	D63.1, E08.2 – E08.29, E10.2 – E10.29, E11.2 – E11.29, E12.2, E13.2 – E13.29, E14.2, I12 – I13.9, N02 – N08.8, N15.0, N17 – N19, Q60 – Q63.2, Q63.8 – Q63.9, Q64.2 – Q64.9	D63.1, E08.2 – E08.29, E10.2 – E10.29, E11.2 – E11.29, E12.2, E13.2 – E13.29, E14.2, I12 – I13.9, N02 – N08.8, N15.0, N17 – N19, P96.0 – P96.0	249.4 – 249.41, 250.4 – 250.49, 285.21, 403 – 404.93, 581 – 587.9, 753.0 – 753.4, 753.6 – 753.9, V13.03 – V13.09, V18.6, V18.69, V42.0, V45.1 – V45.12, V45.73, V56 – V56.8, V59.4, V81.5 – V81.6	249.4 – 249.41, 250.4 – 250.49, 285.21, 403 – 404.93, 581 – 587.9
4	Chronic kidney disease due to type 1 diabetes mellitus	-	-	250.4, 250.41, 250.43 – 250.49	-

Level	Cause	ICD-10	ICD-10 used in hospital/Claims analyses	ICD-9	ICD-9 used in hospital/Claims analyses
4	Chronic kidney disease due to type 2 diabetes mellitus	-	-	250.40, 250.42	-
4	Chronic kidney disease due to hypertension	I12 – I13.9	-	403 – 404.93	-
4	Chronic kidney disease due to glomerulonephritis	N03 – N06.9, N08 – N08.8	-	581 – 583.9	-
4	Chronic kidney disease due to other and unspecified causes	N02 – N02.9, N07 – N07.9, Q60 – Q63.2, Q63.8 – Q63.9, Q64.2 – Q64.9	-	753.0 – 753.4, 753.6 – 753.9	-
**2**	**Other non-communicable diseases**	-	-	-	-
3	Congenital birth defects	-	-	-	-
4	Congenital heart anomalies	Q20 – Q27, Q27.1 – Q28.9	Q20 – Q28.9	745 – 747.9	745 – 747.9
3	Hemoglobinopathies and hemolytic anemias	–	–	–	–
4	Thalassemias	D56 – D56.3, D56.5 – D56.9, D57.4 – D57.419	D56 – D56.9	282.4 – 282.49, 282.6 – 282.62, V78, V78.2 – V78.9	282.4, 282.44 – 282.49, V78 – V78.9
4	G6PD deficiency	D55 – D55.2	D55 – D55.2	282.3	282.3
4	Other hemoglobinopathies and hemolytic anemias	D55.3 – D55.9, D56.4, D58 – D61.9, D64 – D64.8	D55.3 – D55.9, D58 – D61.9, D64 – D64.8	282 – 282.2, 282.7 – 285.0, 285.8 – 285.9	282 – 282.2, 282.7 – 285.0, 285.8 – 285.9
4	Thyroid diseases	E03 – E03.1, E03.3 – E06.3, E06.5 – E07, E07.1, P72.1	E03 – E03.1, E03.3 –E06.3, E06.5 – E07, E07.1, P72.1	240 – 243.9, 245 – 246.9, 775.3	240 – 243.9, 245 – 246.9, 775.3

GBD Global Burden of Diseases, *ICD* International Classification of Diseases

Table S3 Number, rate, and age-standardized prevalence rate of heart failure, and percentage change from 1990 to 2023 by severity and sex

Severity	Sex	Number in millions (95% UI)		Percentage change, 1990 – 2023 [% (95%CI)]	Prevalence rate per 100,000 population (95% UI)		Percentage change, 1990 – 2023 [% (95%CI)]	Age-standardized prevalence rate [per 100,000 population (95% UI)]		Percentage change, 1990 – 2023 [% (95%CI)]
		1990	2023		1990	2023		1990	2023	
Heart failure	Both	4.6 (4.1 – 5.2)	14.3 (12.3 – 16.4)	208.4 (188.5 – 224.4)	392.7 (345.1 – 444.8)	998.4 (859.4 – 1145.2)	154.2 (137.8 – 167.4)	618.7 (544.6 – 696.5)	677.0 (591.5 – 766.2)	9.4 (4.1 – 14.1)
	Male	2.4 (2.1 – 2.8)	7.5 (6.4 – 8.6)	209.9 (189.1 – 227.5)	398.8 (349.2 – 453.2)	1025.6 (880.3 – 1179)	157.2 (139.9 – 171.7)	687.3 (603.8 – 777.7)	750.2 (652.6 – 847)	9.2 (3.4 – 14.6)
	Female	2.2 (1.9 – 2.5)	6.8 (5.8 – 7.8)	206.8 (188.2 – 224)	386.3 (339.1 – 436.4)	969.9 (836.8 – 1112.7)	151.1 (135.9 – 165.1)	558.4 (488.4 – 626.4)	608.1 (529.7 – 688)	8.9 (3.7 – 14)
Treated heart failure	Both	1.7 (1.5 – 2)	5.2 (4.5 – 6.1)	208.4 (188.9 – 224.6)	144.1 (125.4 – 166.1)	366.2 (314.2 – 428.2)	154.2 (138.1 – 167.5)	227 (198.6 – 262)	248.3 (215.5 – 286.1)	9.4 (4.1 – 14)
	Male	0.9 (0.8 – 1)	2.8 (2.4 – 3.2)	209.9 (189 – 228.1)	146.3 (128 – 168.3)	376.2 (323.3 – 440.3)	157.1 (139.8 – 172.2)	252.1 (218.1 – 289.9)	275.2 (239.5 – 315.8)	9.1 (3.4 – 14.5)
	Female	0.8 (0.7 – 0.9)	2.5 (2.1 – 2.9)	206.8 (188.5 – 224)	141.7 (123 – 164.3)	355.8 (304.7 – 417.5)	151 (136.1 – 165.2)	204.9 (178.9 – 235.6)	223.1 (193.1 – 257.2)	8.9 (3.7 – 14.1)
Mild heart failure	Both	0.9 (0.7 – 1.1)	2.7 (2.0 – 3.5)	208.6 (187.8 – 225.1)	73.3 (55.4 – 92.6)	186.5 (137.2 – 246.3)	154.3 (137.2 – 167.9)	115.5 (86.3 – 147.8)	126.4 (95.1 – 165)	9.5 (3.9 – 14.3)
	Male	0.5 (0.3 – 0.6)	1.4 (1.0 – 1.8)	210 (187.1 – 227.9)	74.5 (56.5 – 94.1)	191.6 (139.8 – 251.5)	157.3 (138.2 – 172.1)	128.3 (95.8 – 165)	140.1 (104.8 – 181.5)	9.2 (3.3 – 14.7)
	Female	0.4 (0.3 – 0.5)	1.3 (0.9 – 1.7)	207 (186.4 – 224.5)	72.1 (54.2 – 91.1)	181.2 (133.8 – 242.3)	151.2 (134.4 – 165.5)	104.3 (78.6 – 134.2)	113.6 (86 – 149.1)	8.9 (3.8 – 14.1)

Severity	Sex	Number in millions (95% UI)		Percentage change, 1990 – 2023 [% (95%CI)]	Prevalence rate per 100,000 population (95% UI)		Percentage change, 1990 – 2023 [% (95%CI)]	Age-standardized prevalence rate [per 100,000 population (95% UI)]		Percentage change, 1990 – 2023 [% (95%CI)]
		1990	2023		1990	2023		1990	2023	
Moderate heart failure	Both	0.6 (0.4 – 0.7)	1.7 (1.3 – 2.3)	208.4 (189.4 – 224.8)	47.7 (36.2 – 61.8)	121.2 (90.8 – 158.5)	154.2 (138.6 – 167.7)	75.1 (56.8 – 98.3)	82.2 (62.5 – 106.6)	9.5 (3.7 – 14.2)
	Male	0.3 (0.2 – 0.4)	0.9 (0.7 – 1.2)	209.9 (189.6 – 227)	48.4 (36.6 – 63.2)	124.5 (92.9 – 163.1)	157.2 (140.3 – 171.4)	83.4 (62.6 – 109.1)	91.1 (68.7 – 117.6)	9.2 (3.2 – 14.7)
	Female	0.3 (0.2 – 0.3)	0.8 (0.6 – 1.1)	206.8 (187.6 – 225.2)	46.9 (35.3 – 60.7)	117.8 (88.5 – 11.4)	151.1 (135.4 – 166.1)	67.8 (51.7 – 89)	73.8 (56.4 – 96.3)	8.9 (3.5 – 14.5)
Severe heart failure	Both	1.5 (1.3 – 1.7)	4.6 (3.9 – 5.4)	208.4 (189.4 – 225.2)	127.6 (107.1 – 147.5)	324.4 (270.9 – 379.1)	154.2 (138.5 – 168)	201.1 (169.7 – 233.5)	220 (185.5 – 255.1)	9.4 (4.2 – 14.1)
	Male	0.8 (0.7 – 0.9)	2.4 (2 – 2.8)	209.9 (189.7 – 226.7)	129.6 (108.7 – 150.5)	333.3 (276.7 – 388.2)	157.1 (140.4 – 171.1)	223.4 (187.9 – 260)	243.8 (205.2 – 282.1)	9.1 (3.3 – 14.6)
	Female	0.7 (0.6 – 0.8)	2.2 (1.8 – 2.6)	206.8 (188.6 – 224.1)	125.6 (105.5 – 145.7)	315.2 (261.4 – 372)	151 (136.2 – 165.2)	181.5 (153.1 – 210.8)	197.6 (166.4 – 230.1)	8.9 (3.7 – 13.9)

UI uncertainty interval

Table S4 Number, rate, and age-standardized YLDs rate of heart failure, and percentage change from 1990 to 2023 by severity and sex

Severity	Sex	Number in thousands (95% UI)		Percentage change, 1990 – 2023 [% (95%CI)]	YLDs rate per 100,000 population (95% UI)		Percentage change, 1990 – 2023 [% (95%CI)]	Age-standardized YLDs rate [per 100,000 population (95% UI)]		Percentage change, 1990 – 2023 [% (95%CI)]
		1990	2023		1990	2023		1990	2023	
Heart failure	Both	455.2 (309.3 – 632.1)	1399.9 (958.1 – 1987.6)	207.5 (189.5 – 223.1)	38.6 (26.2 – 53.6)	97.8 (67 – 138.9)	153.4 (138.6 – 166.3)	60.5 (41.2 – 84.2)	66.0 (45.6 – 93.1)	9.1 (4 – 13.7)
	Male	237.9 (162.8 – 328.9)	740.6 (506 – 1043.2)	211.3 (191.5 – 228.1)	39.1 (26.8 – 54.1)	101.1 (69.1 – 142.5)	158.4 (141.9 – 172.3)	67.1 (46.2 – 92.8)	73.5 (50.8 – 103.1)	9.6 (4.1 – 14.7)
	Female	217.3 (147 – 301.6)	659.2 (450 – 944.4)	203.3 (185.4 – 220.6)	38.0 (25.7 – 52.8)	94.4 (64.4 – 135.2)	148.2 (133.5 – 162.4)	54.7 (37.3 – 76.4)	59.0 (40.6 – 83.8)	7.8 (2.7 – 12.7)
Treated heart failure	Both	122.1 (84.7 – 169.7)	372.4 (255.6 – 531.1)	204.9 (186.2 – 222.7)	10.4 (7.2 – 14.4)	26.0 (17.9 – 37.1)	151.3 (135.9 – 166)	16.4 (11.4 – 22.7)	17.3 (12 – 24.6)	5.1 (–0.9 to 10.2)
	Male	63.2 (44 – 87.3)	199.5 (135.2 – 280.9)	215.5 (196.4 – 234.2)	10.4 (7.2 – 14.4)	27.2 (18.5 – 38.4)	161.8 (145.9 – 177.3)	18.1 (12.5 – 24.9)	19.5 (13.5 – 27.5)	7.8 (1.1 – 13)
	Female	58.9 (40.2 – 81.7)	172.9 (119.4 – 249.6)	193.4 (175 – 212.8)	10.3 (7 – 14.3)	24.8 (17.1 – 35.7)	140.1 (125 – 156)	14.9 (10.3 – 20.7)	15.2 (10.5 – 21.9)	1.8 (–4.8 to 7.3)
Moderate heart failure	Both	39.1 (24.9 – 58.2)	120.7 (75.6 – 181.8)	208.7 (187.8 – 225.4)	3.3 (2.1 – 4.9)	8.4 (5.3 – 12.7)	154.4 (137.2 – 168.2)	5.2 (3.3 – 7.7)	5.7 (3.6 – 8.6)	10.4 (4.6 – 15.7)
	Male	20.5 (13 – 30.6)	63.6 (39.8 – 95.6)	210.3 (187.1 – 229.4)	3.4 (2.1 – 5)	8.7 (5.4 – 13.1)	157.5 (138.3 – 173.3)	5.8 (3.7 – 8.5)	6.4 (4 – 9.6)	10.2 (3.3 – 16.2)
	Female	18.6 (11.9 – 27.6)	57.1 (35.8 – 86.3)	207.0 (187.0 – 225.4)	3.3 (2.1 – 4.8)	8.2 (5.1 – 12.4)	151.2 (134.9 – 166.3)	4.7 (3 – 7)	5.1 (3.2 – 7.8)	9.9 (4.1 – 15.4)

Severity	Sex	Number in thousands (95% UI)		Percentage change, 1990 – 2023 [% (95%CI)]	YLDs rate per 100,000 population (95% UI)		Percentage change, 1990 – 2023 [% (95%CI)]	Age-standardized YLDs rate [per 100,000 population (95% UI)]		Percentage change, 1990 – 2023 [% (95%CI)]
		1990	2023		1990	2023		1990	2023	
Mild heart failure	Both	35.0 (19.4 – 55.8)	108.2 (60.5 – 176.1)	208.8 (188.6 – 227)	3.0 (1.6 – 4.7)	7.6 (4.2 – 12.3)	154.5 (137.9 – 169.5)	4.7 (2.6 – 7.5)	5.1 (2.9 – 8.3)	10.3 (4.9 – 15.7)
	Male	18.4 (10.2 – 29.1)	57.0 (31.7 – 92)	210.3 (189.8 – 229.9)	3.0 (1.7 – 4.8)	7.8 (4.3 – 12.6)	157.5 (140.5 – 173.8)	5.2 (2.9 – 8.3)	5.7 (3.3 – 9.2)	10.0 (3.9 – 15.6)
	Female	16.7 (9.2 – 26.7)	51.3 (28.8 – 83.8)	207.2 (185.3 – 225.3)	2.9 (1.6 – 4.7)	7.3 (4.1 – 12)	151.4 (133.5 – 166.2)	4.2 (2.3 – 6.8)	4.6 (2.6 – 7.5)	9.8 (4 – 15.4)
Severe heart failure	Both	258.9 (176.3 – 366.2)	798.5 (530.8 – 1125.6)	208.4 (188.9 – 225.9)	22.0 (15 – 31.1)	55.8 (37.1 – 78.7)	154.2 (138.1 – 168.6)	34.3 (23.4 – 47.8)	37.9 (25.7 – 53.4)	10.7 (5.4 – 15.4)
	Male	135.8 (91.6 – 191.9)	420.6 (281.5 – 596.7)	209.7 (188.9 – 228.6)	22.3 (15.1 – 31.6)	57.4 (38.4 – 81.5)	157 (139.7 – 172.6)	38.1 (26.1 – 52.9)	42.0 (28.4 – 59.2)	10.4 (4.5 – 16)
	Female	123.1 (83.8 – 172.7)	377.9 (251.2 – 534.6)	207 (187.1 – 225.1)	21.5 (14.7 – 30.2)	54.1 (36 – 76.5)	151.2 (134.9 – 166)	30.9 (20.9 – 43.3)	34.0 (22.9 – 47.9)	10.2 (4.9 – 15.3)

UI uncertainty interval

Table S5 Number (in thousands) and proportion of overall heart failure by cause in China in 1990 and 2023 [number in thousands(%)]

**Cause**	**1990**	**2023**
Hypertensive heart disease	1531.72 (33.08)	4299.35 (30.1)
Ischemic heart disease	1154.12 (24.92)	5404.24 (37.84)
Congenital heart anomalies	393.61 (8.5)	450.41 (3.15)
Chronic obstructive pulmonary disease	377.15 (8.14)	1352.99 (9.47)
Rheumatic heart disease	349.64 (7.55)	471.36 (3.3)
Intracerebral hemorrhage	189.93 (4.1)	522.52 (3.66)
Non-rheumatic degenerative mitral valve disease	138.7 (3)	362.76 (2.54)
Other cardiomyopathy	91.39 (1.97)	270.03 (1.89)
Ischemic stroke	85.24 (1.84)	360.84 (2.53)
Chronic kidney disease due to other and unspecified causes	48.17 (1.04)	119.76 (0.84)
Myocarditis	41.05 (0.89)	40.26 (0.28)
Subarachnoid hemorrhage	33.7 (0.73)	41.61 (0.29)
Chronic hepatitis B including cirrhosis	24.07 (0.52)	41.34 (0.29)
Atrial fibrillation and flutter	23.02 (0.5)	149.75 (1.05)
Pulmonary arterial hypertension	22.12 (0.48)	41.61 (0.29)
Non-rheumatic calcific aortic valve disease	19.01 (0.41)	83.74 (0.59)
Chronic kidney disease due to diabetes mellitus type 2	17.15 (0.37)	73.37 (0.51)
Thalassemias	15.86 (0.34)	12.88 (0.09)
Other cardiovascular and circulatory diseases	12.3 (0.27)	22.59 (0.16)
Chronic kidney disease due to hypertension	10.14 (0.22)	40.09 (0.28)
Endocarditis	10.13 (0.22)	13.6 (0.1)
Chronic kidney disease due to glomerulonephritis	9.14 (0.2)	19.62 (0.14)

Cause	1990	2023
Alcoholic cardiomyopathy	5.66 (0.12)	28.45 (0.2)
Other hemoglobinopathies and hemolytic anemias	5.56 (0.12)	9.79 (0.07)
Thyroid diseases	4.7 (0.1)	7.53 (0.05)
Amphetamine use disorders	4.57 (0.1)	4.4 (0.03)
Chronic hepatitis C including cirrhosis	3.4 (0.07)	5.7 (0.04)
Cirrhosis due to other causes	2.83 (0.06)	2.13 (0.01)
G6PD deficiency	2.19 (0.05)	3.24 (0.02)
Cirrhosis due to alcohol	1.45 (0.03)	3.94 (0.03)
Silicosis	0.83 (0.02)	7.37 (0.05)
Nonalcoholic fatty liver disease including cirrhosis	0.73 (0.02)	2.34 (0.02)
Cocaine use disorders	0.44 (0.01)	0.34 (0)
Interstitial lung disease and pulmonary sarcoidosis	0.43 (0.01)	9.71 (0.07)
Chronic kidney disease due to diabetes mellitus type 1	0.3 (0.01)	0.85 (0.01)
Other non-rheumatic valve diseases	0.27 (0.01)	1.47 (0.01)
Coal workers pneumoconiosis	0.15 (0)	0.55 (0)
Other pneumoconiosis	0.11 (0)	0.64 (0)
Asbestosis	0.02 (0)	0.24 (0)
Chagas disease	0 (0)	0 (0)

Table S6 Number (in thousands) and proportion of treated heart failure by cause in China in 1990 and 2023 [number in thousands(%)]

Cause	1990	2023
Hypertensive heart disease	561.99 (33.08)	1577.43 (30.11)
Ischemic heart disease	423.17 (24.91)	1981.48 (37.82)
Congenital heart anomalies	144.42 (8.5)	165.27 (3.15)
Chronic obstructive pulmonary disease	138.26 (8.14)	496.04 (9.47)
Rheumatic heart disease	128.21 (7.55)	172.84 (3.3)
Intracerebral hemorrhage	69.84 (4.11)	192.15 (3.67)
Non-rheumatic degenerative mitral valve disease	50.92 (3)	133.16 (2.54)
Other cardiomyopathy	33.51 (1.97)	99.01 (1.89)
Ischemic stroke	31.34 (1.84)	132.69 (2.53)
Chronic kidney disease due to other and unspecified causes	17.67 (1.04)	43.92 (0.84)
Myocarditis	15.06 (0.89)	14.77 (0.28)
Subarachnoid hemorrhage	12.39 (0.73)	15.3 (0.29)
Chronic hepatitis B including cirrhosis	8.83 (0.52)	15.16 (0.29)
Atrial fibrillation and flutter	8.44 (0.5)	54.9 (1.05)
Pulmonary arterial hypertension	8.12 (0.48)	15.27 (0.29)
Non-rheumatic calcific aortic valve disease	6.98 (0.41)	30.74 (0.59)
Chronic kidney disease due to diabetes mellitus type 2	6.29 (0.37)	26.9 (0.51)
Thalassemias	5.82 (0.34)	4.73 (0.09)
Other cardiovascular and circulatory diseases	4.51 (0.27)	8.29 (0.16)
Endocarditis	3.72 (0.22)	4.99 (0.1)
Chronic kidney disease due to hypertension	3.72 (0.22)	14.7 (0.28)
Chronic kidney disease due to glomerulonephritis	3.35 (0.2)	7.2 (0.14)

Cause	1990	2023
Alcoholic cardiomyopathy	2.07 (0.12)	10.43 (0.2)
Other hemoglobinopathies and hemolytic anemias	2.04 (0.12)	3.59 (0.07)
Thyroid diseases	1.73 (0.1)	2.76 (0.05)
Amphetamine use disorders	1.68 (0.1)	1.61 (0.03)
Chronic hepatitis C including cirrhosis	1.25 (0.07)	2.09 (0.04)
Cirrhosis due to other causes	1.04 (0.06)	0.78 (0.01)
G6PD deficiency	0.8 (0.05)	1.19 (0.02)
Cirrhosis due to alcohol	0.53 (0.03)	1.44 (0.03)
Silicosis	0.3 (0.02)	2.7 (0.05)
Nonalcoholic fatty liver disease including cirrhosis	0.27 (0.02)	0.86 (0.02)
Interstitial lung disease and pulmonary sarcoidosis	0.16 (0.01)	3.56 (0.07)
Cocaine use disorders	0.16 (0.01)	0.13 (0)
Chronic kidney disease due to diabetes mellitus type 1	0.11 (0.01)	0.31 (0.01)
Other non-rheumatic valve diseases	0.1 (0.01)	0.54 (0.01)
Coal workers pneumoconiosis	0.06 (0)	0.2 (0)
Other pneumoconiosis	0.04 (0)	0.23 (0)
Asbestosis	0.01 (0)	0.09 (0)
Chagas disease	0 (0)	0 (0)

Table S7 Number (in thousands) and proportion of mild heart failure by cause in China in 1990 and 2023 [number in thousands(%)]

Cause	1990	2023
Hypertensive heart disease	285.7 (33.04)	802.01 (30.06)
Ischemic heart disease	215.93 (24.97)	1011.04 (37.89)
Congenital heart anomalies	73.31 (8.48)	83.88 (3.14)
Chronic obstructive pulmonary disease	70.48 (8.15)	252.75 (9.47)
Rheumatic heart disease	65.39 (7.56)	88.22 (3.31)
Intracerebral hemorrhage	35.44 (4.1)	97.47 (3.65)
Non-rheumatic degenerative mitral valve disease	25.85 (2.99)	67.72 (2.54)
Other cardiomyopathy	17.1 (1.98)	50.49 (1.89)
Ischemic stroke	15.9 (1.84)	67.31 (2.52)
Chronic kidney disease due to other and unspecified causes	8.99 (1.04)	22.37 (0.84)
Myocarditis	7.66 (0.89)	7.52 (0.28)
Subarachnoid hemorrhage	6.29 (0.73)	7.76 (0.29)
Chronic hepatitis B including cirrhosis	4.5 (0.52)	7.73 (0.29)
Atrial fibrillation and flutter	4.31 (0.5)	27.99 (1.05)
Pulmonary arterial hypertension	4.09 (0.47)	7.68 (0.29)
Non-rheumatic calcific aortic valve disease	3.55 (0.41)	15.66 (0.59)
Chronic kidney disease due to diabetes mellitus type 2	3.21 (0.37)	13.72 (0.51)
Thalassemias	2.93 (0.34)	2.38 (0.09)
Other cardiovascular and circulatory diseases	2.3 (0.27)	4.21 (0.16)
Chronic kidney disease due to hypertension	1.9 (0.22)	7.49 (0.28)
Endocarditis	1.89 (0.22)	2.54 (0.1)
Chronic kidney disease due to glomerulonephritis	1.71 (0.2)	3.67 (0.14)

Cause	1990	2023
Alcoholic cardiomyopathy	1.06 (0.12)	5.32 (0.2)
Other hemoglobinopathies and hemolytic anemias	1.03 (0.12)	1.81 (0.07)
Thyroid diseases	0.88 (0.1)	1.4 (0.05)
Amphetamine use disorders	0.86 (0.1)	0.82 (0.03)
Chronic hepatitis C including cirrhosis	0.64 (0.07)	1.07 (0.04)
Cirrhosis due to other causes	0.53 (0.06)	0.4 (0.01)
G6PD deficiency	0.4 (0.05)	0.6 (0.02)
Cirrhosis due to alcohol	0.27 (0.03)	0.74 (0.03)
Silicosis	0.15 (0.02)	1.38 (0.05)
Nonalcoholic fatty liver disease including cirrhosis	0.14 (0.02)	0.44 (0.02)
Interstitial lung disease and pulmonary sarcoidosis	0.08 (0.01)	1.82 (0.07)
Cocaine use disorders	0.08 (0.01)	0.06 (0)
Chronic kidney disease due to diabetes mellitus type 1	0.06 (0.01)	0.16 (0.01)
Other non-rheumatic valve diseases	0.05 (0.01)	0.28 (0.01)
Coal workers pneumoconiosis	0.03 (0)	0.1 (0)
Other pneumoconiosis	0.02 (0)	0.12 (0)
Asbestosis	0 (0)	0.04 (0)
Chagas disease	0 (0)	0 (0)

Table S8 Number (in thousands) and proportion of moderate heart failure by cause in China in 1990 and 2023 [number in thousands(%)]

Cause	1990	2023
Hypertensive heart disease	186.13 (33.1)	522.67 (30.14)
Ischemic heart disease	140.05 (24.91)	656.07 (37.83)
Congenital heart anomalies	47.88 (8.51)	54.78 (3.16)
Chronic obstructive pulmonary disease	45.8 (8.15)	164.23 (9.47)
Rheumatic heart disease	42.45 (7.55)	57.22 (3.3)
Intracerebral hemorrhage	22.92 (4.08)	63.07 (3.64)
Non-rheumatic degenerative mitral valve disease	16.82 (2.99)	43.99 (2.54)
Other cardiomyopathy	11.09 (1.97)	32.77 (1.89)
Ischemic stroke	10.29 (1.83)	43.56 (2.51)
Chronic kidney disease due to other and unspecified causes	5.85 (1.04)	14.55 (0.84)
Myocarditis	4.98 (0.89)	4.88 (0.28)
Subarachnoid hemorrhage	4.07 (0.72)	5.02 (0.29)
Chronic hepatitis B including cirrhosis	2.92 (0.52)	5.02 (0.29)
Atrial fibrillation and flutter	2.8 (0.5)	18.2 (1.05)
Pulmonary arterial hypertension	2.73 (0.49)	5.14 (0.3)
Non-rheumatic calcific aortic valve disease	2.3 (0.41)	10.14 (0.58)
Chronic kidney disease due to diabetes mellitus type 2	2.08 (0.37)	8.92 (0.51)
Thalassemias	1.96 (0.35)	1.59 (0.09)
Other cardiovascular and circulatory diseases	1.49 (0.27)	2.74 (0.16)
Endocarditis	1.23 (0.22)	1.65 (0.1)
Chronic kidney disease due to hypertension	1.23 (0.22)	4.87 (0.28)
Chronic kidney disease due to glomerulonephritis	1.11 (0.2)	2.38 (0.14)

Cause	1990	2023
Other hemoglobinopathies and hemolytic anemias	0.69 (0.12)	1.21 (0.07)
Alcoholic cardiomyopathy	0.69 (0.12)	3.46 (0.2)
Thyroid diseases	0.57 (0.1)	0.92 (0.05)
Amphetamine use disorders	0.55 (0.1)	0.53 (0.03)
Chronic hepatitis C including cirrhosis	0.41 (0.07)	0.69 (0.04)
Cirrhosis due to other causes	0.34 (0.06)	0.26 (0.01)
G6PD deficiency	0.27 (0.05)	0.4 (0.02)
Cirrhosis due to alcohol	0.18 (0.03)	0.48 (0.03)
Silicosis	0.1 (0.02)	0.89 (0.05)
Nonalcoholic fatty liver disease including cirrhosis	0.09 (0.02)	0.28 (0.02)
Interstitial lung disease and pulmonary sarcoidosis	0.05 (0.01)	1.18 (0.07)
Cocaine use disorders	0.05 (0.01)	0.04 (0)
Chronic kidney disease due to diabetes mellitus type 1	0.04 (0.01)	0.1 (0.01)
Other non-rheumatic valve diseases	0.03 (0.01)	0.18 (0.01)
Coal workers pneumoconiosis	0.02 (0)	0.07 (0)
Other pneumoconiosis	0.01 (0)	0.08 (0)
Asbestosis	0 (0)	0.03 (0)
Chagas disease	0 (0)	0 (0)

Table S9 Number (in thousands) and proportion of severe heart failure by cause in China in 1990 and 2023 [number in thousands(%)]

Cause	1990	2023
Hypertensive heart disease	497.89 (33.08)	1397.24 (30.1)
Ischemic heart disease	374.97 (24.91)	1755.65 (37.82)
Congenital heart anomalies	128.01 (8.5)	146.48 (3.16)
Chronic obstructive pulmonary disease	122.6 (8.15)	439.97 (9.48)
Rheumatic heart disease	113.59 (7.55)	153.08 (3.3)
Intracerebral hemorrhage	61.73 (4.1)	169.83 (3.66)
Non-rheumatic degenerative mitral valve disease	45.11 (3)	117.89 (2.54)
Other cardiomyopathy	29.69 (1.97)	87.75 (1.89)
Ischemic stroke	27.7 (1.84)	117.28 (2.53)
Chronic kidney disease due to other and unspecified causes	15.65 (1.04)	38.91 (0.84)
Myocarditis	13.34 (0.89)	13.09 (0.28)
Subarachnoid hemorrhage	10.95 (0.73)	13.52 (0.29)
Chronic hepatitis B including cirrhosis	7.82 (0.52)	13.43 (0.29)
Atrial fibrillation and flutter	7.48 (0.5)	48.66 (1.05)
Pulmonary arterial hypertension	7.19 (0.48)	13.52 (0.29)
Non-rheumatic calcific aortic valve disease	6.18 (0.41)	27.21 (0.59)
Chronic kidney disease due to diabetes mellitus type 2	5.57 (0.37)	23.84 (0.51)
Thalassemias	5.15 (0.34)	4.18 (0.09)
Other cardiovascular and circulatory diseases	4 (0.27)	7.35 (0.16)
Endocarditis	3.29 (0.22)	4.42 (0.1)
Chronic kidney disease due to hypertension	3.29 (0.22)	13.03 (0.28)
Chronic kidney disease due to glomerulonephritis	2.97 (0.2)	6.38 (0.14)

Cause	1990	2023
Alcoholic cardiomyopathy	1.84 (0.12)	9.24 (0.2)
Other hemoglobinopathies and hemolytic anemias	1.81 (0.12)	3.18 (0.07)
Thyroid diseases	1.53 (0.1)	2.45 (0.05)
Amphetamine use disorders	1.48 (0.1)	1.43 (0.03)
Chronic hepatitis C including cirrhosis	1.11 (0.07)	1.85 (0.04)
Cirrhosis due to other causes	0.92 (0.06)	0.69 (0.01)
G6PD deficiency	0.71 (0.05)	1.05 (0.02)
Cirrhosis due to alcohol	0.47 (0.03)	1.28 (0.03)
Silicosis	0.27 (0.02)	2.39 (0.05)
Nonalcoholic fatty liver disease including cirrhosis	0.24 (0.02)	0.76 (0.02)
Interstitial lung disease and pulmonary sarcoidosis	0.14 (0.01)	3.16 (0.07)
Cocaine use disorders	0.14 (0.01)	0.11 (0)
Chronic kidney disease due to diabetes mellitus type 1	0.1 (0.01)	0.28 (0.01)
Other non-rheumatic valve diseases	0.09 (0.01)	0.48 (0.01)
Coal workers pneumoconiosis	0.05 (0)	0.18 (0)
Other pneumoconiosis	0.04 (0)	0.21 (0)
Asbestosis	0.01 (0)	0.08 (0)
Chagas disease	0 (0)	0 (0)

Table S10 Top 10 causes of heart failure by all-age prevalence rate and proportion in all causes in 2023 and EAPC in prevalence rate in China, 1990 – 2023

Cause	Prevalence rate (per 100,000 population)	Proportion in all – cause (%)	EAPC in prevalence rate [% (95% UI)]
All causes	998.39	100	2.93(2.70 – 3.17) *
1 Ischemic heart disease	377.75	37.84	4.55(4.17 – 4.95) *
2 Hypertensive heart disease	300.52	30.1	2.47(2.04 – 2.93) *
3 Chronic obstructive pulmonary disease	94.57	9.47	2.95(2.43 – 3.47) *
4 Intracerebral hemorrhage	36.52	3.66	2.19(1.58 – 2.81) *
5 Rheumatic heart disease	32.95	3.30	− 0.06(− 0.43 to 0.30)
6 Congenital heart anomalies	31.48	3.15	0.13(− 0.20 to 0.47)
7 Non-rheumatic degenerative mitral valve disease	25.36	2.54	2.34(1.46 – 3.31) *
8 Ischemic stroke	25.22	2.53	3.80(3.12 – 4.51) *
9 Other cardiomyopathies	18.87	1.89	3.24(2.86 – 3.65) *
10 Atrial fibrillation and flutter	10.47	1.05	4.99(4.40 – 5.60) *

*Indicates a significant increase or decrease. EAPC values are presented with two decimal places for greater clarity due to their relatively small magnitude.
EAPC estimated annual percentage change, *UI* uncertainty interval

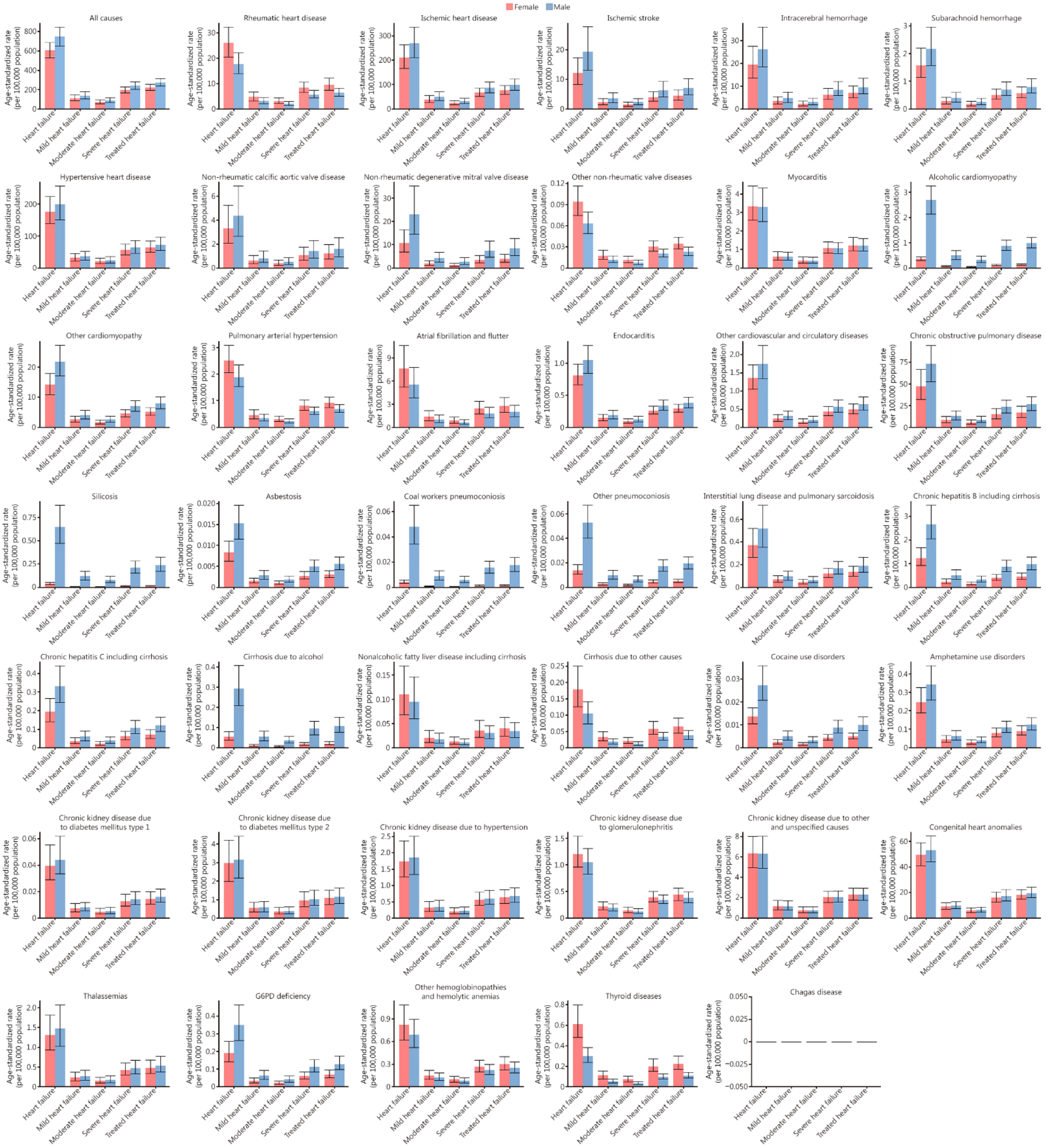

Fig. S1 Age-standardized prevalence of heart failure due to 40 causes by sex, 2023

a

Underlying causes 1990	Underlying causes 2023	EAPC in numbers of all age YLDs number (95% UI)	EAPC in age-standardized YLDs rate per 100,000 population (95% UI)
1 Hypertensive heart disease	1 Ischemic heart disease	5.34(4.61–6.15)	1.72(1.03–2.45)
2 Ischemic heart disease	2 Hypertensive heart disease	3.12(2.37–3.88)	−0.54(−1.27 to 0.22)
3 Chronic obstructive pulmonary disease	3 Chronic obstructive pulmonary disease	3.37(2.67–4.09)	−0.44(−1.11 to 0.20)
4 Congenital heart anomalies	4 Intracerebral hemorrhage	3.20(2.42–4.04)	−0.47(−1.19 to 0.31)
5 Intracerebral hemorrhage	5 Ischemic stroke	4.79(3.96–5.67)	0.83(0.04–1.67)
6 Rheumatic heart disease	6 Congenital heart anomalies	0.60(−0.11 to 1.39)	1.37(0.65–2.16)
7 Ischemic stroke	7 Non-rheumatic degenerative mitral valve disease	3.12(2.06–4.27)	−0.97(−1.95 to 0.08)
8 Non-rheumatic degenerative mitral valve disease	8 Rheumatic heart disease	0.61(−0.13 to 1.35)	−1.84(−2.54 to −1.14)
9 Other cardiomyopathy	9 Other cardiomyopathy	3.57(2.78–4.40)	2.45(1.69–3.23)
10 Chronic kidney disease due to other and unspecified causes	10 Chronic kidney disease due to other and unspecified causes	2.57(1.79–3.36)	0.49(−0.24 to 1.24)
11 Subarachnoid hemorrhage	11 Atrial fibrillation and flutter	6.51(5.52–7.51)	1.95(1.01–2.94)
12 Chronic hepatitis B including cirrhosis	12 Subarachnoid hemorrhage	0.93(0.18–1.72)	−2.41(−3.07 to −1.67)
13 Myocarditis	13 Chronic kidney disease due to diabetes mellitus type 2	4.61(3.70–5.59)	1.09(0.22–2.00)
14 Chronic kidney disease due to diabetes mellitus type 2	14 Chronic hepatitis B including cirrhosis	1.47(0.69–2.26)	−1.13(−1.85 to −0.35)
15 Non-rheumatic calcific aortic valve disease	15 Non-rheumatic calcific aortic valve disease	4.71(0.00 to 0.00)	0.63(0.00 to 0.00)
16 Pulmonary Arterial Hypertension	16 Chronic kidney disease due to hypertension	4.35(3.48–5.25)	0.87(0.04–1.77)
17 Thalassemias	17 Alcoholic cardiomyopathy	6.42(5.60–7.30)	4.62(3.85–5.45)
18 Other cardiovascular and circulatory diseases	18 Myocarditis	−0.24(−0.90 to 0.47)	−0.66(−1.36 to 0.04)
19 Atrial fibrillation and flutter	19 Pulmonary Arterial Hypertension	2.19(1.45–2.99)	0.49(−0.21 to 1.26)
20 Chronic kidney disease due to hypertension	20 Other cardiovascular and circulatory diseases	1.54(0.74–2.40)	0.59(−0.22 to 1.45)
21 Endocarditis	21 Chronic kidney disease due to glomerulonephritis	2.04(1.30–2.86)	0.67(−0.06 to 1.49)
22 Chronic kidney disease due to glomerulonephritis	22 Endocarditis	−0.73(−1.49 to 0.04)	−1.59(−2.33 to −0.83)
23 Alcoholic cardiomyopathy	23 Thalassemias	−0.53(−1.44 to 0.44)	0.24(−0.69 to 1.22)
24 Chronic hepatitis C including cirrhosis	24 Silicosis	6.98(6.12–7.85)	3.60(2.77–4.49)
25 Other hemoglobinopathies and hemolytic anemias	25 Chronic hepatitis C including cirrhosis	1.90(1.02–2.80)	−0.84(−1.68 to 0.04)
26 Amphetamine use disorders	26 Cirrhosis due to alcohol	3.35(2.40–4.32)	0.24(−0.67 to 1.20)
27 Cirrhosis due to alcohol	27 Interstitial lung disease and pulmonary sarcoidosis	10.09(9.15–11.04)	6.40(5.51–7.34)
28 Cirrhosis due to other causes	28 Other hemoglobinopathies and hemolytic anemias	1.86(1.05–2.74)	1.07(0.26–1.94)
29 G6PD deficiency	29 Amphetamine use disorders	−1.00(−1.75 to −0.22)	−1.84(−2.58 to −1.08)
30 Thyroid diseases	30 Thyroid diseases	1.90(1.18–2.64)	0.45(−0.23 to 1.15)
31 Silicosis	31 G6PD deficiency	1.34(0.56–2.23)	1.52(0.71–2.43)
32 Nonalcoholic fatty liver disease including cirrhosis	32 Nonalcoholic fatty liver disease including cirrhosis	3.72(2.72–4.72)	0.55(−0.38 to 1.55)
33 Interstitial lung disease and pulmonary sarcoidosis	33 Cirrhosis due to other causes	−0.92(−1.77 to −0.00)	−1.63(−2.45 to −0.75)
34 Cocaine use disorders	34 Chronic kidney disease due to diabetes mellitus type 1	2.81(1.92–3.69)	0.72(−0.09 to 1.58)
35 Chronic kidney disease due to diabetes mellitus type 1	35 Other non-rheumatic valve diseases	3.97(3.16–4.81)	1.55(0.75–2.36)
36 Other non-rheumatic valve diseases	36 Other pneumoconiosis	5.01(4.23–5.80)	2.58(1.85–3.32)
37 Coal workers pneumoconiosis	37 Coal workers pneumoconiosis	3.66(2.79–4.56)	0.14(−0.71 to 1.04)
38 Other pneumoconiosis	38 Cocaine use disorders	−1.70(−2.50 to −0.82)	−2.57(−3.38 to −1.71)
39 Asbestosis	39 Asbestosis	6.79(5.99–7.59)	3.90(3.13–4.69)
40 Chagas disease	40 Chagas disease	0.00(0.00 to 0.00)	0.00(0.00 to 0.00)

b

Underlying causes 1990	Underlying causes 2023	EAPC in numbers of all age YLDs number (95% UI)	EAPC in age-standardized YLDs rate per 100,000 population (95% UI)
1 Hypertensive heart disease	1 Ischemic heart disease	4.98(4.28–5.73)	1.36(0.68–2.05)
2 Ischemic heart disease	2 Hypertensive heart disease	3.05(2.31–3.82)	−0.53(−1.22 to 0.20)
3 Intracerebral hemorrhage	3 Chronic obstructive pulmonary disease	3.87(2.98–4.84)	−0.02(−0.85 to 0.87)
4 Rheumatic heart disease	4 Intracerebral hemorrhage	2.44(1.67–3.26)	−1.07(−1.80 to −0.28)
5 Congenital heart anomalies	5 Ischemic stroke	4.00(3.20–4.86)	0.25(−0.51 to 1.06)
6 Chronic obstructive pulmonary disease	6 Rheumatic heart disease	0.46(−0.26 to 1.17)	−2.11(−2.81 to −1.43)
7 Ischemic stroke	7 Congenital heart anomalies	0.87(0.16–1.62)	1.63(0.92–2.38)
8 Non-rheumatic degenerative mitral valve disease	8 Non-rheumatic degenerative mitral valve disease	2.66(1.62–3.82)	−1.20(−2.17 to −0.13)
9 Subarachnoid hemorrhage	9 Other cardiomyopathy	4.24(3.45–5.06)	2.90(2.12–3.68)
10 Other cardiomyopathy	10 Atrial fibrillation and flutter	5.11(4.12–6.19)	0.53(−0.42 to 1.53)
11 Chronic kidney disease due to other and unspecified causes	11 Chronic kidney disease due to other and unspecified causes	2.90(2.12–3.70)	0.70(−0.03 to 1.46)
12 Myocarditis	12 Chronic kidney disease due to diabetes mellitus type 2	4.48(3.55–5.52)	1.10(0.22–2.09)
13 Chronic hepatitis B including cirrhosis	13 Subarachnoid hemorrhage	−1.64(−2.36 to −0.88)	−4.83(−5.49 to −4.10)
14 Atrial fibrillation and flutter	14 Non-rheumatic calcific aortic valve disease	4.51(0.00 to 0.00)	0.47(0.00 to 0.00)
15 Pulmonary Arterial Hypertension	15 Chronic kidney disease due to hypertension	4.19(3.32–5.08)	0.89(0.05–1.78)
16 Chronic kidney disease due to diabetes mellitus type 2	16 Pulmonary Arterial Hypertension	2.05(1.29–2.84)	0.18(−0.57 to 0.95)
17 Non-rheumatic calcific aortic valve disease	17 Chronic hepatitis B including cirrhosis	0.58(−0.24 to 1.41)	−2.29(−3.04 to −1.49)
18 Chronic kidney disease due to hypertension	18 Myocarditis	0.60(−0.09 to 1.38)	0.27(−0.46 to 1.01)
19 Thalassemias	19 Chronic kidney disease due to glomerulonephritis	2.61(1.87–3.43)	1.09(0.36–1.86)
20 Chronic kidney disease due to glomerulonephritis	20 Other cardiovascular and circulatory diseases	2.30(1.48–3.14)	1.23(0.43–2.06)
21 Other cardiovascular and circulatory diseases	21 Endocarditis	1.16(0.44–1.93)	0.16(−0.55 to 0.90)
22 Thyroid diseases	22 Thalassemias	−0.06(−0.96 to 0.93)	0.68(−0.24 to 1.70)
23 Endocarditis	23 Other hemoglobinopathies and hemolytic anemias	1.77(0.94–2.65)	1.30(0.47–2.16)
24 Other hemoglobinopathies and hemolytic anemias	24 Thyroid diseases	0.77(0.07–1.53)	−0.54(−1.26 to 0.18)
25 Cirrhosis due to other causes	25 Interstitial lung disease and pulmonary sarcoidosis	12.03(11.12–12.97)	8.78(7.91–9.71)
26 Chronic hepatitis C including cirrhosis	26 Chronic hepatitis C including cirrhosis	1.31(0.42–2.23)	−1.67(−2.53 to −0.79)
27 Amphetamine use disorders	27 Alcoholic cardiomyopathy	5.61(4.90–6.41)	3.61(2.90–4.35)
28 G6PD deficiency	28 Cirrhosis due to other causes	−0.84(−1.67 to 0.04)	−1.98(−2.76 to −1.13)
29 Alcoholic cardiomyopathy	29 Nonalcoholic fatty liver disease including cirrhosis	3.50(2.44–4.61)	0.16(−0.83 to 1.26)
30 Nonalcoholic fatty liver disease including cirrhosis	30 Amphetamine use disorders	−1.03(−1.79 to −0.25)	−1.66(−2.42 to −0.89)
31 Cirrhosis due to alcohol	31 Cirrhosis due to alcohol	2.36(1.35–3.36)	−0.96(−1.93 to −0.00)
32 Chronic kidney disease due to diabetes mellitus type 1	32 G6PD deficiency	1.01(0.18–1.94)	1.33(0.46–2.26)
33 Cocaine use disorders	33 Other non-rheumatic valve diseases	6.55(5.74–7.41)	4.51(3.72–5.34)
34 Interstitial lung disease and pulmonary sarcoidosis	34 Chronic kidney disease due to diabetes mellitus type 1	3.36(2.44–4.31)	1.05(0.23–1.96)
35 Other non-rheumatic valve diseases	35 Silicosis	10.33(9.46–11.19)	6.98(6.16–7.87)
36 Silicosis	36 Other pneumoconiosis	7.09(6.31–7.99)	5.28(4.54–6.10)
37 Other pneumoconiosis	37 Cocaine use disorders	−1.70(−2.40 to −0.99)	−3.20(−3.88 to −2.49)
38 Coal workers pneumoconiosis	38 Asbestosis	8.66(7.83–9.48)	6.31(5.53–7.08)
39 Asbestosis	39 Coal workers pneumoconiosis	5.69(4.84–6.56)	2.90(2.12–3.73)
40 Chagas disease	40 Chagas disease	0.00(0.00 to 0.00)	0.00(0.00 to 0.00)

Fig. S2 Heart failure due to underlying causes rankings by absolute number of YLDs in males (**a**) and females (**b**) in China, 1990 – 2023. YLDs years lived with disability

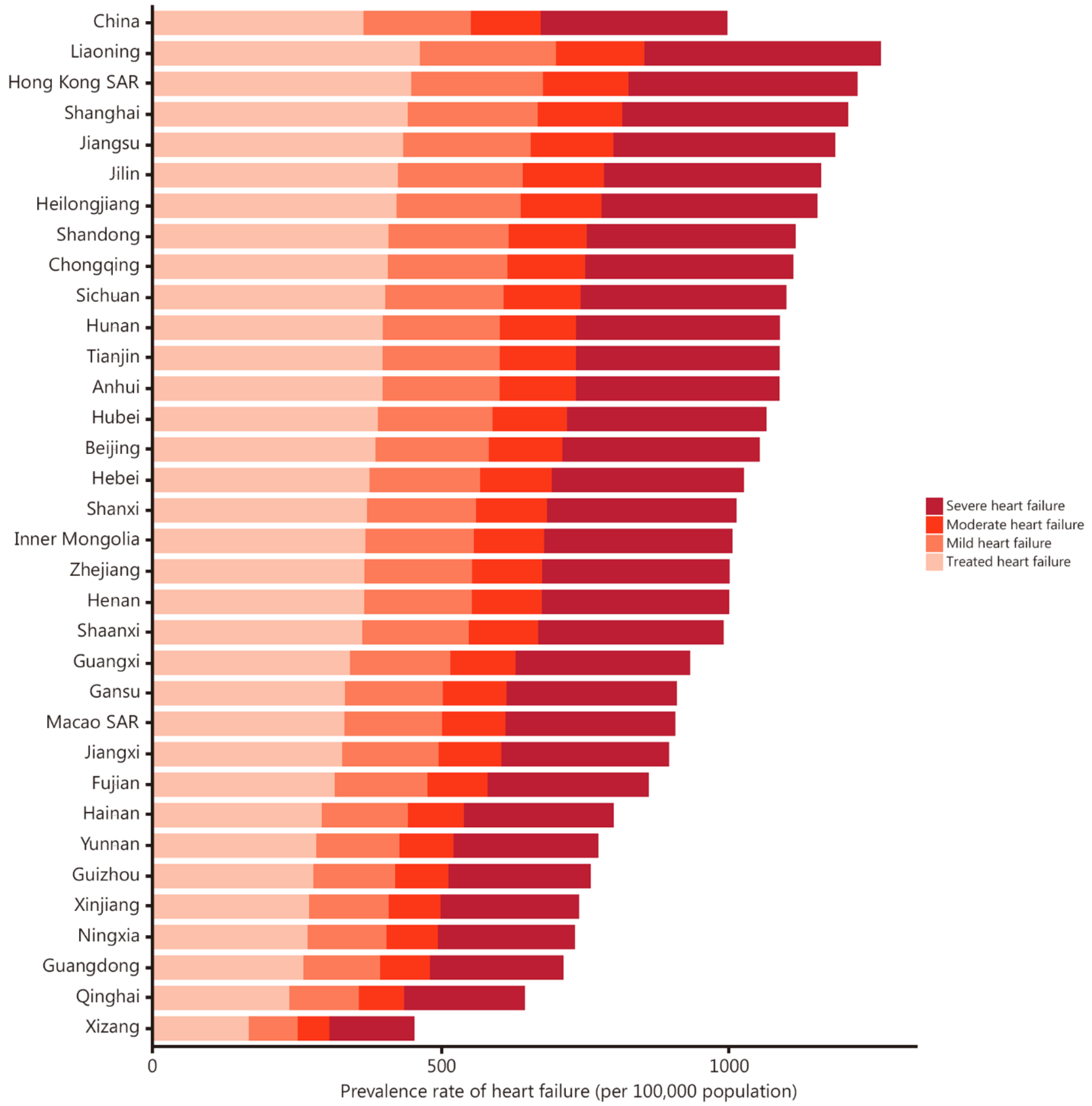

Fig. S3 Prevalence rate of heart failure (per 100,000 population) in China, 2023

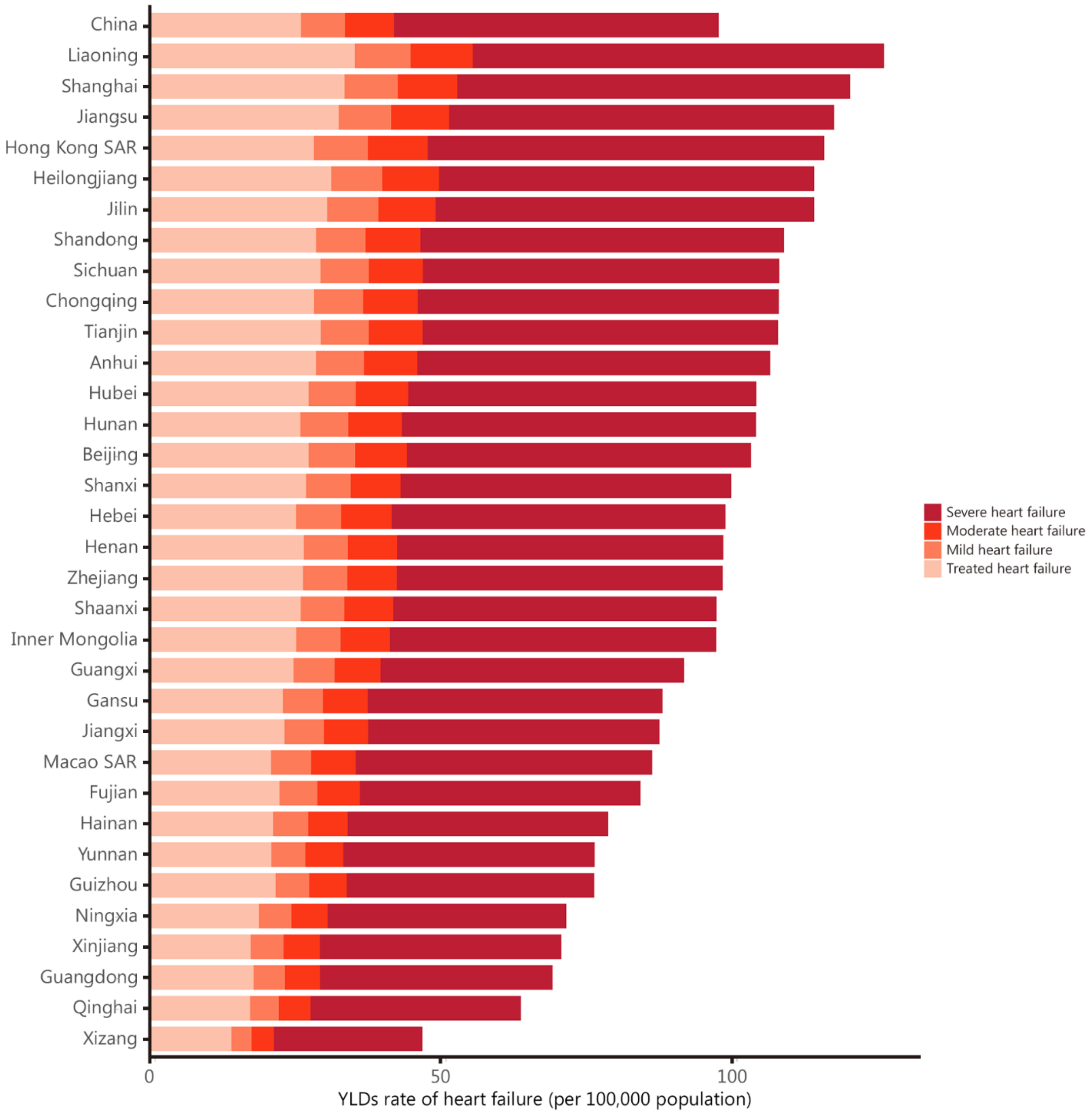

Fig. S4 YLDs rate of heart failure (per 100,000 population) in China, 2023. YLDs years lived with disability

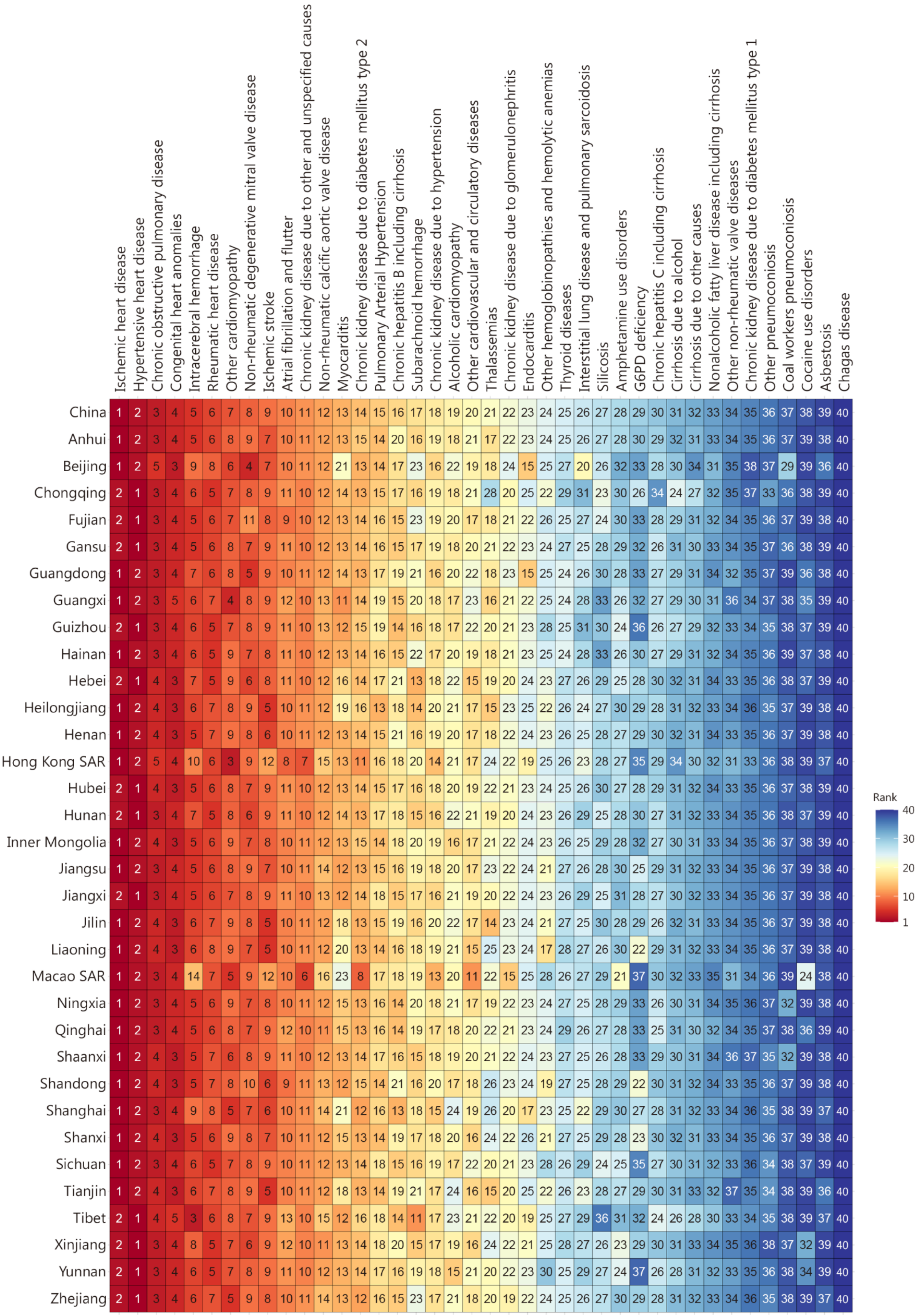

Fig. S5 Age-standardized prevalence rate of heart failure due to 40 causes in China, 2023

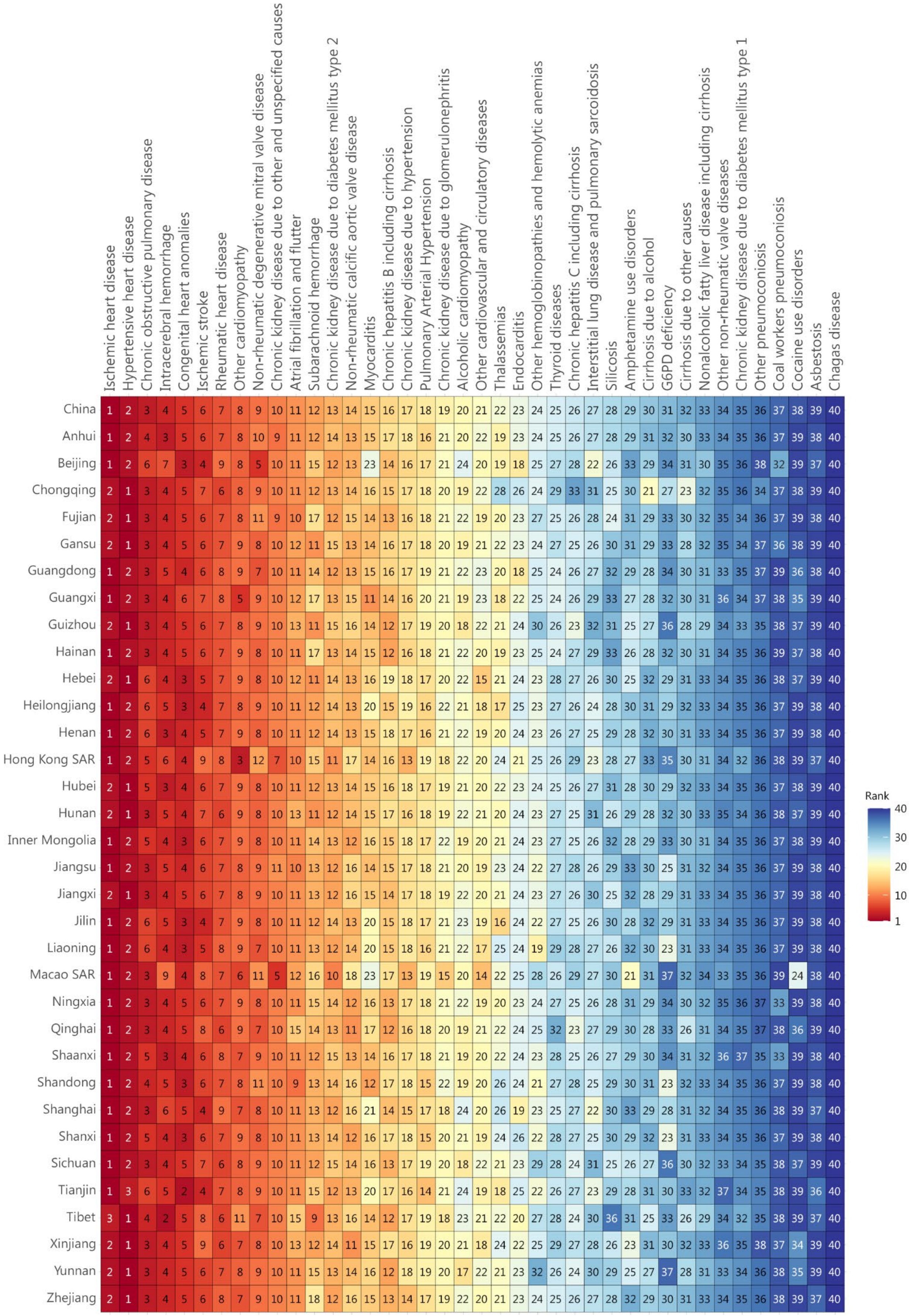

Fig. S6 Age-standardized YLDs rate of heart failure due to 40 causes in China, 2023. YLDs years lived with disability

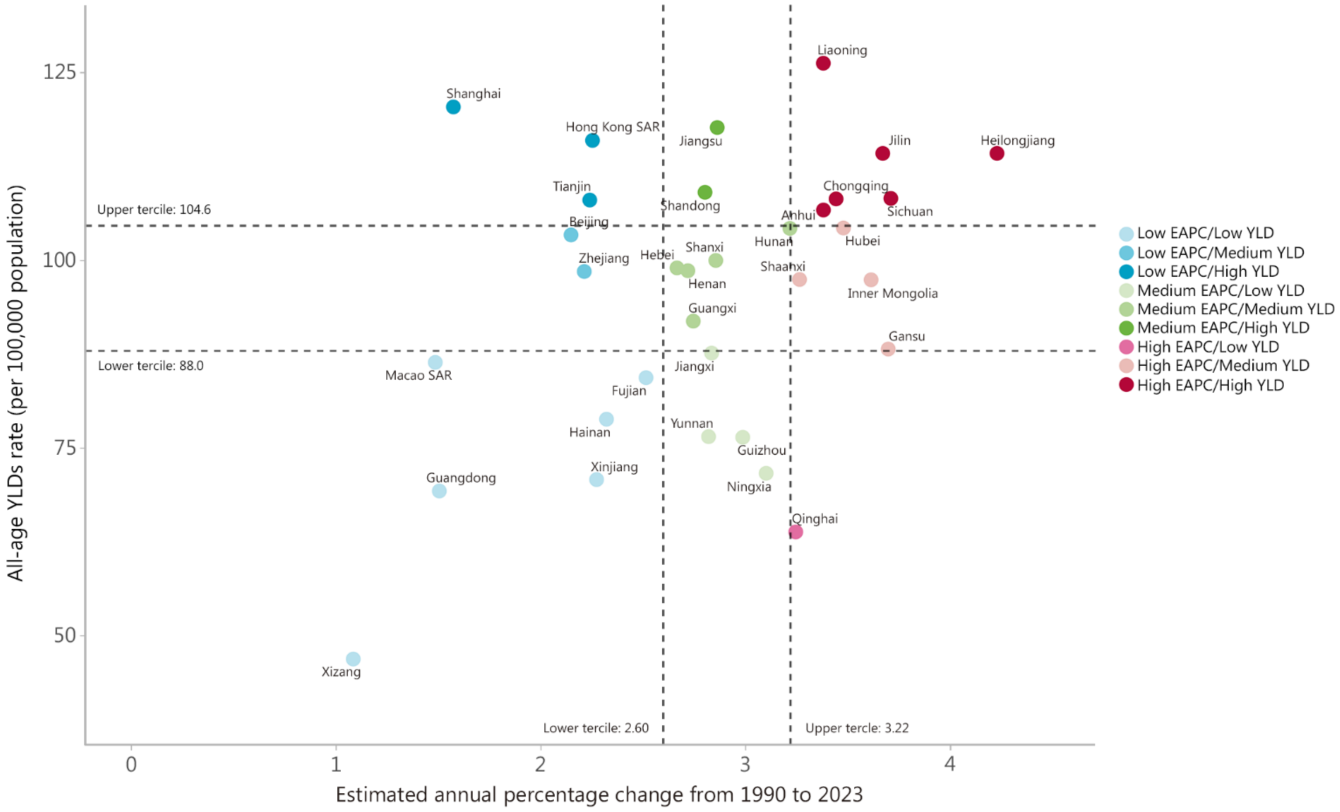

Fig. S7 All-age YLDs rates per 100,000 population in 2023 and estimated annual percentage change in YLDs rates per 100,000 population in 1990 – 2023 due to heart failure for all ages and both sexes in China. YLDs years lived with disability
